# Influenza Vaccine-Induced Antibody Responses Are Not Impaired by Frailty in the Community-Dwelling Elderly With Natural Influenza Exposure

**DOI:** 10.3389/fimmu.2018.02465

**Published:** 2018-10-24

**Authors:** Vipin Narang, Yanxia Lu, Crystal Tan, Xavier F. N. Camous, Shwe Zin Nyunt, Christophe Carre, Esther Wing Hei Mok, Glenn Wong, Sebastian Maurer-Stroh, Brian Abel, Nicolas Burdin, Michael Poidinger, Paul Anantharajah Tambyah, Nabil Bosco, Lucian Visan, Tze Pin Ng, Anis Larbi

**Affiliations:** ^1^Singapore Immunology Network, Singapore, Singapore; ^2^Department of Psychological Medicine, National University Hospital, Singapore, Singapore; ^3^Sanofi Pasteur, Marcy-l'Étoile, France; ^4^Bioinformatics Institute, Singapore, Singapore; ^5^Division of Infectious Diseases, National University Hospital, Singapore, Singapore; ^6^Nestlé Research Singapore Hub, Singapore, Singapore

**Keywords:** frailty, influenza vaccine, immune response, antibody response, elderly

## Abstract

**Background:** Elderly adults over 65 years of age are recommended to receive seasonal influenza vaccination as they are at a higher risk of infection and its complications than the younger community. The elderly are often stratified according to frailty status where frail individuals are more susceptible to adverse health outcomes than their non-frail counterparts, however, it is not known whether immunity induced by influenza vaccination is impaired in the frail elderly.

**Study Design:** Two hundred and five elderly subjects of Chinese ethnicity in Singapore (mean age 73.3 ± 5.3 years, 128 females and 77 males) were administered the recommended trivalent inactivated 2013–14 seasonal influenza vaccine (Vaxigrip™) containing A/H1N1, A/H3N2, and B strains. The elderly subjects were stratified into three groups according to Fried's frailty criteria (59 frail, 85 pre-frail, 61 robust) and were also ranked by Rockwood's frailty index (RFI). Statistical associations were evaluated between frailty status and pre- and post-vaccination antibody titres in sera measured by Hemagglutination inhibition (HAI) and microneutralization (MN) assays. Immunological responses across frailty strata were also studied in terms of leukocyte cellular distribution, cytokine levels and gene expression.

**Results**: Post-vaccination, 83.4% of the subjects seroconverted for A/H1N1, 80.5% for A/H3N2, and 81% for the B strain. The seroconversion rates were comparable across frailty groups (A/H1N1, ANOVA, *p* = 0.7910; A/H3N2, ANOVA, *p* = 0.8356, B, ANOVA, *p* = 0.9741). Geometric mean titres of HAI and MN as well as seroprotection rates were also similar in all three frailty groups and uncorrelated with RFI (Spearman, *r* = 0.023, *p* = 0.738). No statistically significant differences were observed between the frailty groups in vaccine-induced modulation of leukocyte populations, cytokine responses, and gene expression profiles of peripheral blood mononuclear cells (PBMCs). Whereas, post- and pre-vaccination HAI titres were positively correlated after adjusting for age and gender (A/H1N1, *R*^2^ = 0.216, *p* = 9.1e−11; A/H3N2, *R*^2^ = 0.166, *p* = 3.4e−8; B, *R*^2^ = 0.104, *p* = 3.1e−5). With most subjects lacking previous history of influenza vaccination, the pre-vaccination titres were likely due to natural exposure and seen to match the pattern of influenza subtype prevalence in the time period of vaccination.

**Conclusion**: The majority of the elderly subjects seroconverted for seasonal influenza upon vaccination, and importantly, influenza vaccination-induced humoral immune responses and seroprotection were similar across the frailty strata, indicating that frail individuals may also benefit from influenza vaccination. Pre-existing antibodies due to natural exposure appeared to positively influence vaccine-induced antibody responses.

## Introduction

Immune responsiveness declines in the elderly as a consequence of alterations in the distribution and function of immune cells with age and changes induced by chronic viral infections (such as cytomegalovirus) and inflammatory diseases (1, 2). Therefore, elderly individuals above 65 years of age are considered to be at a greater risk of influenza infection and its complications, and it is hence recommended that they receive an annual administration of seasonal influenza vaccine (3).

There is great biological and clinical heterogeneity amongst individuals, which is even further pronounced in the elderly. The concept of frailty was introduced to stratify the elderly population where *frail* individuals pose a higher degree of risk toward disease and mortality as compared to *non-frail* or *robust* individuals (4). Frailty is measured in multiple dimensions including weight loss, weakness, exhaustion, slowness, low physical activity, cognitive impairment, and other health symptoms that would indicate increased vulnerability toward adverse health outcomes (5, 6). Frailty has been shown to influence the course and outcomes of health conditions (7). However, it is not clearly understood whether differences exist between frail and non-frail elderly in their capacity to respond to influenza vaccination, as there are conflicting reports in the literature. While some earlier studies reported reduced humoral responses to influenza vaccine in the frail, (8–10), more recent studies have not supported these findings (11–15). It is important to understand whether frailty has a significant impact on vaccine-induced immunity as this information might guide policy decisions on relevant aspects such as the frequency, dosage and composition of influenza vaccine administered to the elderly and could have an impact on future rational vaccine design strategies.

In this study, immune responses to seasonal influenza vaccination were assessed in an Asian cohort of elderly Chinese Singaporeans stratified by frailty. In addition to assessing the humoral response, which typically comprises the primary endpoint of vaccine responsiveness studies, cell mediated immunity which plays a vital role in immunity toward influenza especially in the elderly (16, 17), markers of innate immune responses, cytokine profiles, and time course transcriptomic profiles of peripheral blood mononuclear cells (PBMCs) were also measured. No significant differences were observed between the frail and non-frail groups in their responsiveness to influenza vaccination in both early and late phases of immune response as well as in the final outcome of virus neutralization.

## Methods

### Recruitment of study participants

A phase IV clinical trial of Sanofi Pasteur's Vaxigrip™ influenza vaccine was approved by the National Healthcare Group's Domain Specific Institutional Review Board and registered at clinicaltrials.gov under the registration number NCT03266237. Older adults above 65 years of age were recruited from December 2013 onwards from participants in the second cohort of Singapore Longitudinal Aging Study (SLAS-2), an epidemiologic study of aging and health as described previously (18, 19). The participants were community dwellers at eight different housing precincts across Singapore. Volunteers were excluded if they had received an influenza vaccine within the 6 months preceding the trial vaccination or planned influenza vaccination during the trial. Those with suspected congenital or acquired immunodeficiency; or in receipt of immunosuppressive therapy such as anti-cancer chemotherapy or radiation therapy within the preceding 6 months; or on long-term systemic corticosteroid therapy (prednisone or equivalent for more than 2 consecutive weeks within the past 3 months) were also excluded. All volunteers provided written informed consent for the administration of seasonal influenza vaccine.

### Frailty measurements

Information on demographic, medical, psychosocial, behavioral, and neurocognitive variables was collected from the study participants at the time of recruitment by trained personnel through interview and on-site clinical assessment. The interview included Instrumental Activities of Daily Living (IADL) (20), Short-form health survey (SF-12) (21), Mini mental state examination (MMSE) (22), geriatric depression scale (GDS) (23), and questions related to occupational, socio-economic, and medical histories and exercise and nutritional habits. Participants self-reported answers to these questionnaires. The on-site clinical assessment included a basic health screening, a 6-meter fast gait speed test, knee extension measurement and Montreal Cognitive Assessment (MoCA) (24). Fried's physical frailty was defined using the five criteria comprising unintentional weight loss, slowness, weakness, exhaustion, and low physical activity (5), which were measured in accordance with an Asian population as described previously (25). In addition, frailty was evaluated on Rockwood's Frailty Index (RFI) (26) by counting an accumulation of 30 deficits each contributing a score between 0 and 1 (Supplementary Figure 1).

### Influenza vaccination

The recommended trivalent inactivated (split virion) Vaxigrip™ (Sanofi Pasteur) 2013–14 seasonal influenza vaccine was used in this trial. The vaccine contained each of the three strains A/California/07/2009 (H1N1), A/Texas/50/2012 (H3N2) and B/Massachusetts/02/2012. Viruses were grown in embryonated chicken eggs, inactivated with formaldehyde, and split with anionic detergent. Vaccine was administered to the 205 elderly study participants between January and August 2014. Venous blood specimens were collected from the participants immediately prior to vaccination (day 0) and on days 2, 7 and 28 after vaccination.

### Vaccine-specific antibody titres

Vaccine-specific antibodies were measured in sera on day 0 and day 28 post-vaccination using the Hemagglutination inhibition (HAI) and microneutralization (MN) assays. For the HAI assay, serum samples were heat inactivated and pretreated with neuraminidase to eliminate nonspecific inhibitors and anti-turkey red blood cell (anti-TRBC) hemagglutinins. The treated serum samples were titrated in 96 well plates starting at a 1/10 dilution and subsequently ten two-fold serial dilutions. Each well was incubated with 4 HA unit/25 μl of the vaccine virus at 37°C for 1 h, followed by the addition of 0.5% TRBC suspension and incubation at ambient temperature for another 1 h. Agglutination was determined by the tilt method. The reciprocal of the highest serum dilution that exhibited complete inhibition of hemagglutination was assigned as the HAI titer.

In the microneutralization assay, the serum samples were again heat-inactivated and two-fold serially diluted in 96 well plates. One-hundred 50% tissue culture infectious doses/50 μl of the vaccine virus was added to each well. After overnight incubation, the wells were washed with 200 μl PBS and the cells were fixed. Infection of the cells was determined by measuring the presence of virus nucleoprotein by enzyme-linked immunosorbent assay (ELISA). The absence of infection of cells indicated successful neutralization due to the presence of influenza virus-specific neutralizing antibodies in the serum. The neutralizing antibody titer was expressed as the reciprocal dilution that caused 50% reduction of the absorbance value in respect of the virus control. This was calculated by the intersection of the neutralization test sample optical density (OD) curve with the line representing the 50% neutralization point of the virus control ODs.

Both HAI and MN assays were performed in two independent runs on each sample and the geometric mean titer of the two runs was used to determine the final titer.

### Luminex

Human cytokine/chemokine panel (Milliplex®, Merck Millipore) was used to measure the levels of IFNγ, IL-6, IL-8, IL-10, IP-10 and TNF-α in plasma samples on days 0, 2, 7, and 28 post-vaccination. Samples and standards were incubated with fluorescent-coded magnetic beads which had been pre-coated with respective capture antibodies. After an overnight incubation at 4°C with shaking, plates were washed twice with wash buffer. Biotinylated detection antibodies were incubated with the complex for 1 h and subsequently Streptavidin-PE was added and incubated for another 30 min. Plates were washed twice again, and beads were re-suspended with sheath fluid in PCR plates before reading on the Luminex analyzer FLEXMAP® 3D. Data was acquired using xPONENT® 4.0 (Luminex®) acquisition software and analyzed using Bio-Plex Manager® 6.1.1 (Bio-Rad). Standard curves generated with a 5PL (5-parameter logistic) algorithm were used for the estimation of MFI and concentration values.

### Immunophenotyping

A Beckman Coulter hematology analyzer (COULTER® Ac·T diff™) was used for counting total red blood cells, total white blood cells, lymphocytes, monocytes, granulocytes, and platelets in whole blood samples. Thereafter, flow cytometry was performed to characterize immune cell subsets. B cells, plasmablasts, CD4 and CD8 T cells, NK cells, and conventional and plasmacytoid dendritic cells were phenotyped in freshly collected whole blood samples. One-hundred μL of whole blood was stained with antibody cocktail (Supplementary Figure 2) in BD Trucount™ Absolute Counting Tubes (BD Biosciences, USA) for 15 min at room temperature. Nine-hundred μL of 1X BD FACS Lysing solution (BD Biosciences, USA) was then added to the tube and incubated for 15 min before acquiring the sample on LSR II Fortessa flow cytometer. Other cell types were phenotyped in cryopreserved PBMCs. Frozen PBMCs were thawed and stained with fluorochrome-conjugated antibodies (Supplementary Figure 2) and FACS analyzed on LSR II Fortessa flow cytometer. Data generated by flow cytometry was analyzed using Flowjo® software (Tree Star, Inc., USA). Events were gated by forward and side scatter and marker expression.

### Microarray

Total RNA was isolated from PBMCs using mirVana™ miRNA isolation kit (Thermo Fisher Scientific, San Jose, CA, USA). Complementary DNA (cDNA) was synthesized using Reverse Transcription Master Mix and Second Strand Master Mix (Thermo Fisher Scientific, San Jose, CA, USA) and purified. Gene expression was assayed using Illumina® human HT-12 V4.0 microarray in batches of 96 samples and the expression data was exported using Illumina® GenomeStudio. Raw expression data was loaded into R/Bioconductor, log_2_ transformed, and normalized using robust spline normalization (RSN) method in lumi package. Only probes which passed a detection *p*-value of 0.05 in 90% of the subjects were retained. The time course data was modeled for differential gene expression analysis using a mixed effect linear model which included subject as a random factor, time point and frailty group as interacting factors, and microarray batch as a fixed factor. The microarray data is available on Gene Expression Omnibus under the accession number GSE107990.

### Statistical methods

HAI titers were analyzed using geometric mean titer (GMT) and geometric standard deviations. The titers were log_2_ transformed and compared across frailty groups by single-factor ANOVA. Following international guidelines (27, 28), seroconversion was defined as either a pre-vaccination HAI titer < 1:10 and a post-vaccination HAI titer > 1:40 or a pre-vaccination HAI titer > 1:10 and a minimum four-fold rise in post-vaccination HAI antibody titer. Seroprotection was defined as achieving an HAI antibody titer ≥ 1:40.

Demographic parameters (age, gender, etc.) and serological history of viral infections (CMV, etc.) were compared across frailty groups by single-factor ANOVA for numerical and Fisher's exact test for categorical data, respectively. Cytokine levels measured by Luminex and counts or percentages of immune cell subsets measured by immunophenotyping assays were compared using the Mann-Whitney-Wilcoxon U test. A paired test was performed when comparing measurements across time points within a frailty group, while an unpaired test was performed when comparing measurements across frailty groups. In all significance tests, *p*-values were adjusted for multiple testing using Benjamini-Hochberg method.

## Results

**Figure 1:**
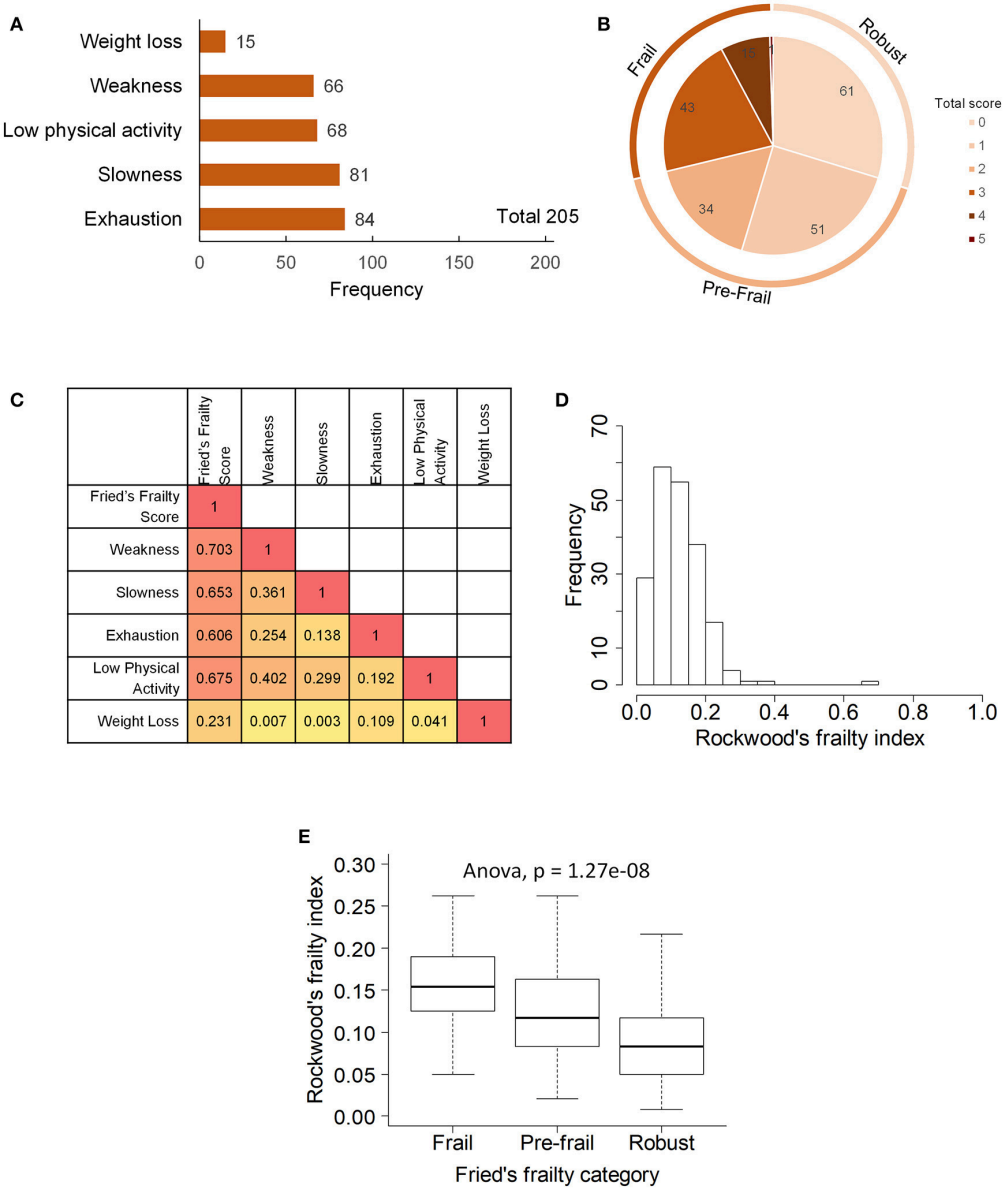
Frailty phenotype in the study participants: **(A)** prevalence of five components of Fried's frailty, **(B)** distribution of Fried's frailty score and definition of frail, pre-frail and robust groups, **(C)** Spearman's rank correlation between frailty components, **(D)** distribution of Rockwood's frailty index, and **(E)** association between Fried's frailty groups and Rockwood's frailty index.

Two hundred and five elderly subjects 65-year-old and above of Chinese ethnicity including 128 (62.4%) females and 77 (37.6%) males were recruited into the study (Table 1). Frailty assessment by Fried's frailty criteria (5) classified 59 subjects as Frail, 85 as Pre-frail and 61 as Robust (Figure 1). Frailty evaluated using Rockwood's Frailty Index (26) correlated well with Fried's frailty categorization (ANOVA *p*-value 1.27e−08). The frail subjects were on average older (*p*-value 0.0048) and had greater leaning toward cognitive decline and depression as indicated by their lower MMSE scores (*p*-value 0.0016) and higher GDS scores (*p*-value 0.003; Table 1).

**Table 1:** Demographic and clinical characteristics of the study cohort across frailty groups.

	**All (*N* = 205)**	**Frail (*N* = 59)**	**Pre-frail (*N* = 85)**	**Robust (*N* = 61)**	***p***	**p.adj**
**SOCIODEMOGRAPHIC**						
Female gender, no. (%)	128 (62.4)	39 (66.1)	54 (63.5)	35 (57.4)	0.5937	0.7050
Age, yr	73.3 ± 5.3	75.1 ± 5.6	72.9 ± 5.1	72.1 ± 5.0	0.0048	0.0304
Age ≥ 75yr, no. (%)	80 (39.0)	31 (52.5)	30 (35.3)	19 (31.1)	0.0403	0.1299
Currently employed, no. (%)	20 (9.8)	5 (8.4)	8 (9.4)	7 (11.4)	0.8726	0.9211
Past or present smoker, no. (%)	40 (19.5)	10 (16.9)	18 (21.2)	12 (19.7)	0.7923	0.8855
Marital (Single/Divorced/Widowed), no. (%)	71 (34.6)	25 (42.4)	30 (35.2)	16 (26.2)	0.1760	0.3646
Living alone	41 (20.0)	13 (22.0)	15 (17.6)	13 (21.3)	0.7764	0.9012
**PHYSICAL HEALTH STATUS**						
BMI, Kg/m^2^	23.7 ± 3.6	22.9 ± 3.8	24.2 ± 3.7	23.8 ± 3.1	0.0841	0.3196
Bone Mineral Density, g/cm^2^						
Spine	0.90 ± 0.19	0.85 ± 0.19	0.90 ± 0.20	0.93 ± 0.16	0.0795	0.2034
Neck	0.76 ± 0.14	0.70 ± 0.15	0.77 ± 0.13	0.78 ± 0.12	0.0038	0.0276
Pelvis	0.94 ± 0.15	0.89 ± 0.15	0.95 ± 0.14	0.97 ± 0.14	0.0062	0.0300
Sarcopenia, no. (%)	82 (40.0)	32 (60.4)	29 (35.8)	21 (37.5)	0.0132	0.0525
Instrumental Activities of Daily Living (IADL)^†^	7.8 ± 1.1	7.6 ± 1.5	7.8 ± 1.2	8.0 ± 0.1	0.1627	0.3630
SF-12 Physical Composite Score (PCS)^†^	46.6 ± 5.4	46.0 ± 6.2	46.1 ± 5.0	47.5 ± 5.2	0.2716	0.4712
Time spent sitting, hours/day	7.1 ± 2.7	9.0 ± 2.5	6.9 ± 2.6	5.4 ± 1.8	5.3e-14	1.5e-12
**COMORBIDITIES**						
High blood pressure, no. (%)	113 (55.1)	29 (49.2)	51 (60.0)	33 (54.1)	0.4370	0.6387
High cholesterol, no. (%)	119 (58.0)	35 (59.3)	49 (57.6)	35 (57.4)	0.9671	0.9671
Diabetes, no. (%)	37 (18.0)	15 (25.4)	16 (18.8)	6 (9.8)	0.0782	0.3196
Arthritis, no. (%)	30 (14.6)	12 (20.3)	11 (12.9)	7 (11.4)	0.3537	0.5260
Poor sleep (PSQI > 5), no. (%)	57 (27.8)	19 (32.2)	24 (28.2)	14 (22.9)	0.3990	0.5260
Comorbidities ≥ 3, no. (%)	63 (30.7)	21 (35.6)	28 (32.9)	14 (22.9)	0.2762	0.4712
**MENTAL HEALTH STATUS**						
Montreal cognitive assessment (MoCA) score	25.2 ± 3.5	24.5 ± 3.9	25.1 ± 3.8	25.9 ± 2.8	0.1890	0.3654
Mini mental state examination (MMSE) score^†^	27.4 ± 2.6	26.4 ± 3.6	27.7 ± 2.1	28.0 ± 2.0	0.0016	0.0285
Geriatric depression scale (GDS) score^§^	0.6 ± 1.2	1.1 ± 1.7	0.5 ± 1.0	0.4 ± 0.8	0.0030	0.0285
Life satisfaction score below 60%, no. (%)	46 (22.4)	16 (27.1%)	24 (28.2)	6 (9.8)	0.0145	0.0526
SF-12 Mental Composite Score (MCS)^†^	49.3 ± 6.2	48.3 ± 6.5	49.6 ± 6.1	49.8 ± 6.0	0.3952	0.6275
**SEROLOGICAL STATUS**						
Cytomegalovirus, positive no. (%)	201 (98)	58 (98.3)	82 (96.5)	61 (100)	0.3063	0.4935
Epstein-Barr virus (EA IgG), positive no. (%)	27 (13.2)	7 (11.9)	11 (12.9)	9 (14.8)	0.5250	0.6650
Epstein-Barr virus (EBNA IgG), positive no. (%)	203 (99)	58 (98.3)	85 (100)	60 (98.4)	0.5122	0.6650
CD4:CD8 T cell ratio (Day 0)	2.42 ± 1.47	2.64 ± 1.76	2.33 ± 1.36	2.32 ± 1.29	0.3963	0.5260

† High score indicates good health;

§ *High score indicates poor health; Values shown are either actual numbers or mean ± sd.*.

Prior to vaccination 27.8% of the subjects were seroprotected against A/H1N1, 54.1% against A/H3N2 and 66.8% against the B strain on the basis of HAI titer measurements of preexisting antibodies in the serum. Pre-vaccination seroprotection rates were similar in the three frailty groups for all three strains (A/H1N1, ANOVA, *p* = 0.4432; A/H3N2, ANOVA, *p* = 0.3776; B, ANOVA, *p* = 0.1545; Table 2). The pre-vaccination HAI titers in terms of geometric mean titers (GMT) were also comparable across the frailty groups (A/H1N1, ANOVA, *p* = 0.7433; A/H3N2, ANOVA, *p* = 0.8734; B, ANOVA, *p* = 0.2713). After vaccination, 83.4% of the subjects seroconverted for A/H1N1, 80.5% for A/H3N2, and 81% for the B strain. The seroconversion rates were comparable across frailty groups (A/H1N1, ANOVA, *p* = 0.7910; A/H3N2, ANOVA, *p* = 0.8356, B, ANOVA, *p* = 0.9741). Post-vaccination seroprotection rates were up to 93.2% against A/H1N1, 97.1% against A/H3N2 and 99.5% against the B strain and were comparable across the three frailty groups (Table 2). The pre- and post-vaccination HAI titers and their ratios and the seroconversion and seroprotection rates also did not correlate with the five individual components of Fried's frailty score (Supplementary Figure 3). RFI showed mild negative correlations with post/pre-vaccination HAI titer ratios (A/H1N1, ρ = −0.029, *r* = −0.026; A/H3N2, ρ = −0.057, *r* = −0.100; B, ρ = −0.108, *r* = −0.139) that were not statistically significant except for Pearson's *r* for the B strain (*p* = 0.0468; Table 3). The mean of titer ratios was >4-fold across the measured range of RFI (0 to 0.7) in a linear regression of titer ratio on RFI (Supplementary Figures 4, 5). In contrast, pre-vaccination HAI titers were significantly correlated with post-vaccination titers (A/H1N1, *R*^2^ = 0.216, *p* = 9.1e-11; A/H3N2, *R*^2^ = 0.166, *p* = 3.4e-8; B, *R*^2^ = 0.104, *p* = 3.1e-5) and post/pre-vaccination titer ratio (A/H1N1, *R*^2^ = 0.265, *p* = 2e-13; A/H3N2, R^2^ = 0.480*, p* < 2e-16; B, *R*^2^ = 0.546*, p* < 2e−16) in a multivariate analysis adjusting for age and gender (Table 4, Supplementary Figure 6). Microneutralization (MN) is used as another specific and sensitive method to measure virus neutralization and has been considered superior to HAI as recently reviewed for influenza vaccines (29). MN assay titers were strongly correlated with HAI titers (A/H1N1, *r*^2^ = 0.93; A/H3N2, *r*^2^ = 0.83; B, *r*^2^ = 0.71, Supplementary Figure 7), and the GMT and seroconversion rates were similar across frailty groups (Table 5).

**Table 2:** Pre- and post-vaccination HAI titres and seroprotection and seroconversion rates across frailty strata.

**Strain**	**HAI responses**	**All (*N* = 205)**	**Frail (*N* = 59)**	**Pre-frail (*N* = 85)**	**Robust (*N* = 61)**	***p***	***p.adj***
A/H1N1	Pre- vaccination (Day 0) HAI titer, GMT	15.3 ± 4.7	13.8 ± 4.8	16.8 ± 4.5	14.7 ± 4.9	0.743	0.95
	Pre-vaccination (Day 0) Seroprotection rate, no. (%)	57 (27.8%)	13 (22%)	27 (31.8%)	17 (27.9%)	0.443	0.95
	Post-vaccination (Day 28) HAI titer, GMT	259 ± 4.7	308.9 ± 5.6	265.3 ± 4.4	211.4 ± 4.5	0.403	0.95
	Post-vaccination (Day 28) Seroprotection rate, no. (%)	191 (93.2%)	56 (94.9%)	80 (94.1%)	55 (90.2%)	0.605	0.95
	Post/Pre-vaccination HAI ratio	17 ± 5	22.4 ± 6	15.8 ± 4.7	14.4 ± 4.3	0.277	0.95
	Seroconversion rate, no. (%)	171 (83.4%)	51 (86.4%)	70 (82.4%)	50 (82%)	0.791	0.95
A/H3N2	Pre-vaccination (Day 0) HAI titer, GMT	52.8 ± 5.8	57.9 ± 6.2	52.1 ± 5.7	49.1 ± 5.8	0.873	0.95
	Pre-vaccination (Day 0) Seroprotection rate, no. (%)	111 (54.1%)	30 (50.8%)	51 (60%)	30 (49.2%)	0.378	0.95
	Post-vaccination (Day 28) HAI titer, GMT	1005.1 ± 3.9	1165.2 ± 3.9	890.5 ± 4.3	1031.4 ± 3.4	0.504	0.95
	Post-vaccination (Day 28) Seroprotection rate, no. (%)	199 (97.1%)	58 (98.3%)	81 (95.3%)	60 (98.4%)	0.585	0.95
	Post/Pre-vaccination HAI ratio	19 ± 5.6	20.1 ± 5.4	17.1 ± 5.4	21 ± 6.3	0.745	0.95
	Seroconversion rate, no. (%)	165 (80.5%)	49 (83.1%)	67 (78.8%)	49 (80.3%)	0.836	0.95
B	Pre-vaccination (Day 0) HAI titer, GMT	67.1 ± 4.6	51.5 ± 5	77.1 ± 4.2	71.4 ± 4.6	0.271	0.95
	Pre-vaccination (Day 0) Seroprotection rate, no. (%)	137 (66.8%)	34 (57.6%)	62 (72.9%)	41 (67.2%)	0.154	0.95
	Post-vaccination (Day 28) HAI titer, GMT	1129.5 ± 3.1	1092.3 ± 3.2	1179.8 ± 2.9	1098 ± 3.1	0.897	0.95
	Post-vaccination (Day 28) Seroprotection rate, no. (%)	204 (99.5%)	59 (100%)	85 (100%)	60 (98.4%)	0.585	0.95
	Post/Pre-vaccination HAI ratio	16.8 ± 4.8	21.2 ± 5.3	15.3 ± 4.3	15.4 ± 5.1	0.411	0.95
	Seroconversion rate, no. (%)	166 (81%)	48 (81.4%)	68 (80%)	50 (82%)	0.974	0.95

**Table 3:** Spearman's ranked correlation between Rockwood frailty index and pre- and post-vaccination HAI titres.

		**Spearman's correlation**			**Pearson's correlation**		
**Strain**	**HAI responses**	**ρ**	***p***	***p.adj***	***r***	***p***	***p.adj***
A/H1N1	Pre-vaccination (Day 0) HAI titer	0.0535	0.446	1	0.0197	0.780	1
	Post-vaccination (Day 28) HAI titer	0.0235	0.738	1	−0.0075	0.915	1
	Post/Pre-vaccination HAI ratio	−0.0290	0.680	1	−0.0263	0.708	1
A/H3N2	Pre-vaccination (Day 0) HAI titer	0.0995	0.156	1	0.0501	0.475	1
	Post-vaccination (Day 28) HAI titer	0.0137	0.846	1	−0.0622	0.375	1
	Post/Pre-vaccination HAI ratio	−0.0565	0.421	1	−0.1000	0.154	1
B	Pre-vaccination (Day 0) HAI titer	0.0239	0.734	1	0.0579	0.4095	1
	Post-vaccination (Day 28) HAI titer	−0.0423	0.547	1	−0.1170	0.0947	0.853
	Post/Pre-vaccination HAI ratio	−0.1076	0.125	1	−0.1390	0.0468	0.468

*The correlations were performed using log_2_ transformed titer values*.

**Table 4:** Multivariate regression models to assess the association of post-vaccination HAI titres or post/pre-vaccination HAI titer ratios with Rockwood frailty score, age, gender and pre-vaccination HAI titres.

**Model:** Post/pre-vaccination HAI ratio ~ Rockwood frailty score + Age + Gender											
**Dependent variable**	**Model statistics**			**Predictor variables**							
	***F***	***p***	**Adjusted *R^2^***	**Age**		**Gender**		**Rockwood frailty score**	
				**β**	***p***	**β**	***p***	**β**	**p**		
HAI, H1N1, Ratio (Day 28/Day 0)	0.699	0.553	−0.004	−0.023	0.484	−0.350	0.309	−0.486	0.831		
HAI, H3N2, Ratio (Day 28 / Day 0)	1.079	0.359	0.001	−0.006	0.866	0.404	0.277	−3.046	0.214		
HAI, B, Ratio (Day 28 / Day 0)	1.775	0.153	0.011	0.003	0.933	−0.383	0.255	–**4.410**	**0.047**		
**Model:** Post-vaccination HAI ~ Rockwood frailty score + Age + Gender + Pre-vaccination HAI											
**Dependent variable**	**Model statistics**			**Predictor variables**							
	***F***	***p***	**Adjusted** ***R**^2^*	**Age**		**Gender**		**Rockwood frailty score**		**HAI, Day 0**	
				β	***p***	β	***p***	β	***p***	β	***p***
HAI, H1N1, Day 28	**15.04**	**9e-11**	**0.216**	−0.017	0.553	−0.480	0.105	−0.348	0.858	**0.463**	**3e-12**
HAI, H3N2, Day 28	**11.17**	**3.4e-8**	**0.166**	0.038	0.142	−0.156	0.565	−2.976	0.093	**0.310**	**4.7e-9**
HAI, B, Day 28	**6.91**	**3.1e-5**	**0.104**	−0.007	0.728	−0.147	0.517	–**2.833**	**0.061**	**0.241**	**1.9e-6**
**Model:** Post/pre-vaccination HAI ratio ~ Rockwood frailty score + Age + Gender + Pre-vaccination HAI											
**Dependent variable**	**Model statistics**			**Predictor variables**							
	***F***	***p***	**Adjusted** ***R**^2^*	**Age**		**Gender**		**Rockwood frailty score**		**HAI, Day 0**	
				β	***P***	β	***p***	β	***p***	β	***p***
HAI, H1N1, Ratio (Day 28 / Day 0)	**19.37**	**2e-13**	**0.265**	−0.017	0.553	−0.480	0.105	−0.348	0.858	–**0.537**	**2e-15**
HAI, H3N2, Ratio (Day 28 / Day 0)	**48.08**	**2e-16**	**0.480**	0.038	0.142	−0.156	0.565	−2.976	0.093	–**0.690**	**2e-16**
HAI, B, Ratio (Day 28 / Day 0)	**62.41**	**2e-16**	**0.546**	−0.007	0.728	−0.147	0.517	**-2.833**	**0.061**	**-0.758**	**2e-16**

*Significant models and predictor variables are shown in bold. The titres were log_2_ transformed*.

**Table 5:** Pre- and post-vaccination microneutralization (MN) titres across frailty strata.

**Strain**	**MN responses**	**All (*N* = 205)**	**Frail (*N* = 59)**	**Pre-frail (*N* = 85)**	**Robust (*N* = 61)**	***p***	***p.adj***
A/H1N1	Pre-vaccination (Day 0) MN titer, GMT	19 ± 5.9	16.8 ± 5.7	21.9 ± 5.7	17.5 ± 6.6	0.6174	0.974
	Post-vaccination (Day 28) MN titer, GMT	477.7 ± 6.3	511.3 ± 8.1	520.4 ± 5.5	397 ± 5.8	0.6453	0.974
	Post/Pre-vaccination MN ratio	25.2 ± 6.1	30.4 ± 7.8	23.7 ± 5.7	22.7 ± 5.1	0.6265	0.974
A/H3N2	Pre-vaccination (Day 0) MN titer, GMT	24.1 ± 3.8	23.3 ± 3.8	26.5 ± 3.7	21.7 ± 4	0.6510	0.974
	Post-vaccination (Day 28) MN titer, GMT	334.4 ± 3.8	339.8 ± 4	318.2 ± 3.9	352.8 ± 3.6	0.8941	0.974
	Post/Pre-vaccination MN ratio	13.9 ± 4.8	14.6 ± 4.1	12 ± 4.7	16.3 ± 5.6	0.4881	0.974
B	Pre-vaccination (Day 0) MN titer, GMT	44.6 ± 4.2	46.2 ± 4.2	42 ± 4.2	47 ± 4.5	0.8802	0.974
	Post-vaccination (Day 28) MN titer, GMT	680.3 ± 4.4	643.2 ± 4.7	707.5 ± 4.3	679.9 ± 4.3	0.9305	0.974
	Post/Pre-vaccination MN ratio	15.2 ± 5.2	13.9 ± 5.3	16.8 ± 4.9	14.5 ± 5.8	0.7644	0.974

**Figure 2:**
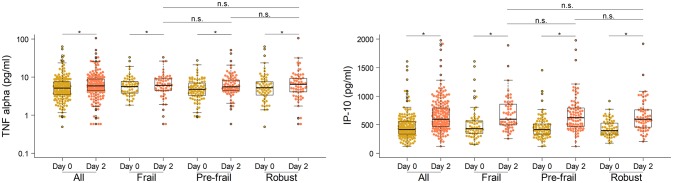
Serum concentrations of TNF-α and IP-10 in vaccine recipients pre- and post-vaccination on day 0 and day 2. *P-value < 0.05, comparing pre- and post-vaccination cytokine levels. The Mann-Whitney-Wilcoxon U test of medians using paired differences was used to determine *p*-values.

**Figure 3:**
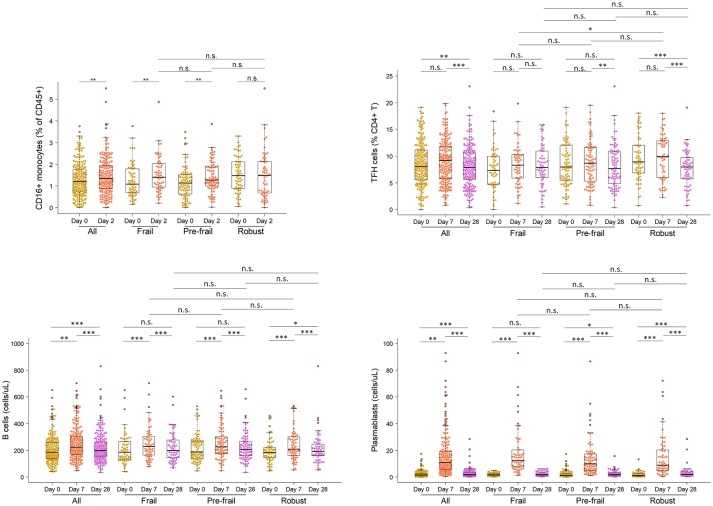
Frequencies of CD16 monocytes, TFH cells, B cells and plasmablasts in vaccine recipients pre- and post-vaccination. *P < 0.05, **P < 0.01, ***P < 0.001. *P*-values comparing pre- and post-vaccination frequencies were determined using Mann-Whitney-Wilcoxon U test of medians using paired differences. *P*-values comparing post-vaccination frequencies between frailty groups were determined using an unpaired Mann-Whitney-Wilcoxon *U*-test of medians.

Next, to comprehensively assess the immunological phenotype across frailty groups, innate and adaptive immune cell subsets and cytokines were analyzed pre- and post-vaccination in peripheral blood samples. Pre-vaccination or baseline distribution of immune cell subsets in the peripheral blood investigated by flow cytometry showed little or no differences between frailty groups (Supplementary Figure 8). Compared to pre-frail and robust, the frail individuals had marginally lower counts of lymphocytes (frail, 1667/μL; pre-frail, 1950/μL; robust, 1975/μL), in particular CD8 T cells (frail, 276/μL; pre-frail, 327/μL; robust, 341/μL), and marginally higher counts of plasmablasts (frail, 1,800/mL; pre-frail, 1,400/mL; robust, 1,200/mL). However, these differences were small and did not alter vaccine immunogenicity as indicated by the measured humoral responses. Notably, on day 2 post-vaccination, the pro-inflammatory cytokines TNF-α and IP-10 (or CXCL10) produced by innate immune responses were significantly induced in similar levels across the three frailty groups (Figure 2). CD16+ monocytes were also present at similar levels on day 2. On day 7 post-vaccination, CD4+ T lymphocytes, in particular follicular T helper cells (T_FH_), which support vaccine induced antibody responses (30), were induced (Figure 3). Although the robust group had slightly higher numbers of T_FH_ as compared to the frail group, the differences were very small in magnitude. Expansion of B cells and antibody secreting plasma cells was evident on day 7 and day 28, however, their frequencies on days 7 and 28 did not differ across frailty groups (Figure 3).

The immunological response to vaccination was also studied using whole genome microarrays to assess gene expression profiles of peripheral blood mononuclear cells pre- and post-vaccination. The assay was performed for 142 subjects in the cohort (frail = 31, pre-frail = 83, robust = 28). Prior to vaccination on day 0, no gene expression differences were observed [zero differentially expressed genes (DEGs)] in pairwise comparisons between the three frailty groups (Table 6). Vaccination led to the modulation of thousands of genes. In comparison to pre-vaccination levels, 5,891 genes were differentially expressed by day 2, 5,065 genes by day 7, and 3,483 genes by day 28, as observed in all 205 elderly subjects. When stratified by frailty, there were greater numbers of DEGs in the pre-frail group as compared to the frail and robust groups. However, this difference may be attributed to larger number of subjects in the pre-frail group as significances generally improve upon increasing the number of replicates in a differential gene expression analysis (31). Therefore, a contrast of contrast analysis was performed to highlight gradient differences in temporal gene expression between frailty groups. In this analysis no significant DEGs were found between the frailty groups until day 7, and this was limited to 2 DEGs between robust and frail groups only. On day 28 there were 271 DEGs between the pre-frail and frail groups, and 174 DEGs between the robust and frail groups with 70 common DEGs between the two contrasts. However, the fold changes were low with no two-fold differential DEGs and gene ontology analysis did not report enrichment of any known biological process or pathway in these DEGs (Supplementary Figure 9). These small numbers (<8% compared to all the genes modulated by vaccination) portray little to no differences between frailty groups in vaccine-induced gene expression modulation.

**Table 6:** Differential gene expression analysis in peripheral blood mononuclear cells of vaccinated subjects.

**Time point**	**Contrast**	**No. of DEGs**
Day 0	Pre-frail vs. Frail	0
	Robust vs. Frail	0
	Robust vs. Pre-frail	0
	Male vs. Female	252
**Group**	**Contrast**	**No. of DEGs**
Frail (*N =* 31)	Day 2 vs. Day 0	531
	Day 7 vs. Day 0	988
	Day 28 vs. Day 0	1186
Pre-frail (*N =* 83)	Day 2 vs. Day 0	4358
	Day 7 vs. Day 0	2954
	Day 28 vs. Day 0	1480
Robust (*N =* 28)	Day 2 vs. Day 0	363
	Day 7 vs. Day 0	1508
	Day 28 vs. Day 0	1022
All elderly (*N =* 142)	Day 2 vs. Day 0	5891
	Day 7 vs. Day 0	5065
	Day 28 vs. Day 0	3483
**Contrast**	**Contrast of contrast**	**No. of DEGs**
Pre-frail vs. Frail	Day 2 vs. Day 0	0
	Day 7 vs. Day 0	0
	Day 28 vs. Day 0	271
Robust vs. Frail	Day 2 vs. Day 0	0
	Day 7 vs. Day 0	2
	Day 28 vs. Day 0	174
Robust vs. Pre-frail	Day 2 vs. Day 0	0
	Day 7 vs. Day 0	0
	Day 28 vs. Day 0	0

*Gene expression was assayed using Illumina HT-12 V4.0 microarrays for 142 out of 205 subjects in the cohort. Differentially expressed genes (DEGs) were shortlisted based on false discovery rate < 0.05*.

The serological history of viral infections, including cytomegalovirus (CMV), Epstein-Barr virus (EBV), H. Pylori, herpes simplex virus (HSV), respiratory syncytial virus (RSV), vesicular stomatitis virus (VSV) and human herpesvirus was measured in the cohort as an independent indicator of immunological status. The proportions of individuals carrying latent viruses or who had been exposed to such viruses was similar in the three frailty groups (Supplementary Figure 10). Thus, no significant immunodeficiency or difference in chronic viral exposure was observed across frailty strata.

## Discussion

Seasonal influenza is a major concern in the elderly as influenza related mortality rises significantly beyond 65 years of age (32). In this study, the elderly subjects responded robustly to influenza vaccination with more than 90% of the individuals attaining seroprotection against all immunized strains as measured by HAI titers. These findings support vaccination as an effective measure for preventing seasonal influenza infection and associated mortality in the elderly.

Among the elderly population, the frail individuals are considered generally more vulnerable to disease and its complications (33). However, whether prophylactic vaccination, such as with the seasonal influenza vaccine, is less immunogenic in the frail is not clear. In this study, no differences were observed between frail and non-frail individuals with respect to pre-existing antibody levels, response to vaccination as indicated by seroconversion rates, as well as post-vaccination seroprotection. In addition, interim analysis of the cellular responses revealed that they were similar in frail and non-frail individuals. Therefore, in this study, frailty does not appear to have an impact on the induction of humoral or cellular immunity after influenza vaccination. A limitation of this study is that the study cohort consisted only of community dwellers and did not include institutionalized subjects and the physically and mentally disabled who might be at the extreme end of frailty or include chronic disease cases. The cohort also had an above average nutritional profile with most individuals scoring 12 or more out of 14 points in mini nutritional assessment (MNA) owing to good socio-economic conditions in Singapore. The low number of extremely frail elderly in this cohort is likely representative of the community dwelling elderly population in Singapore. The cohort in this study was a subset of the Singapore Longitudinal Aging Study (SLAS-2) which included 5,685 community dwelling elderly from south-east, south-west and central parts of Singapore as described in (34), wherein 4.6% of the subjects were categorized as frail as per Fried's frailty criteria. The physical, mental and comorbidity health statuses of the frail elderly in the present cohort were similar to that in the bigger cohort. However, an advantage of looking at relatively healthy elderly is that the results of the clinical trial are not confounded by factors such as the effect of hospitalization. Thus, the high levels of seroconversion and seroprotection in the elderly in this study may be reliable for this population which is a rapidly growing segment of the population in Singapore and most of the high and middle income countries globally.

The results of the present study are in agreement with other recent reports demonstrating a lack of association between frailty and vaccine-induced humoral responses (11, 12, 14, 15), while differing from some of the earlier studies where frailty was associated with lower seroconversion rates (8–10). Of note, the recent studies have suggested that the vaccine-induced antibody production is more strongly correlated with pre-existing antibody levels than with frailty stratification (14, 35). In this study, both pre-existing HAI titers and post-vaccination seroconversion rates were observed to be similar across frailty groups and a positive correlation between pre-existing and post-vaccination antibody titers was seen similarly to other studies (14, 36) confirming that the antibody responses were largely determined by pre-existing antibodies and to a much lesser extent by frailty or age. Most of the participants in this cohort did not have previous history of vaccination. The exclusion criterion of this study excluded individuals who had received influenza vaccine within 6 months prior to the scheduled vaccination and the recruited participants also did not recall having received influenza vaccination within the past 1 year. This reflects the generally low uptake of influenza vaccines in Singapore which is ~15% in all 65 year old and above elderly and even lower in the community dwelling elderly (37) and hence even beyond 1 year most of the participants were unlikely to have been vaccinated. Singapore observes year-round circulation of seasonal influenza due to its geographical location and climate, which might have an impact on influenza exposure and consequently immunological memory and the development of anti-influenza responses. Using official data on circulating influenza strains in Singapore, the baseline titers of different influenza subtypes in the cohort were seen to match the pattern of actual subtype prevalence in the time period of vaccination (Supplementary Figure 11). This data suggests that recent infections by circulating viruses may have largely influenced the baseline titers seen in this cohort.

Antibody responses to influenza vaccination typically tend to be higher in females than in males (38). In this cohort, the female to male ratio was similar in the three frailty groups and therefore gender differences in antibody responses did not influence the association between frailty and antibody response. However, one of the contributing factors to the generally high levels of antibody responses observed in this cohort could be the higher proportion of females in this group. This is generally seen in cohorts of elderly people in most societies, including Singapore, where females tend to have a survival advantage.

Although leukocyte distribution at the baseline and its modulation upon vaccination barely differed between frailty groups, characteristics of immunosenescence were clearly reflected in the elderly in terms of CD4:CD8 T cell ratios above 2 (Table 1) (39) and the accumulation of late stage differentiated T cells, which indicates that, immunosenescence occurs uniformly in the elderly independent of frailty status. Despite immunosenescence, a robust immunological response to vaccination in the elderly may be partly explained by pre-existing anti-influenza immunity induced by regular natural exposure to seasonal influenza associated with long lived memory B and T cells. Robust immunological responses in the elderly have been reported previously (40). However, these findings do not preclude that primary immunological responses to previously unseen pathogens including novel strains of influenza might still be impaired in the elderly.

While this study reports positive immunological outcomes following the administration of preventive influenza vaccination in the elderly across the frailty spectrum, it does not imply that frail and non-frail individuals will respond similarly to pathogenic influenza infection. Upon infection with pathological influenza viruses, disease outcomes may differ between the frail and non-frail groups due to the increased risk of infection and its related complications in the frail and for a number of other reasons. The vaccine formulation, Vaxigrip, used in this study was a subunit vaccine containing minimal amounts of matrix and nucleoprotein which does not elicit sufficient cell-mediated immune responses that would be protective of influenza (41). The data on pre- and post-vaccination counts of CD8 T cells in fact showed no increase in post-vaccination CD8 T cell counts (Supplementary Figure 12). Hence the results of this study may only apply to antibody-mediated protection against influenza and may have limitations as a sole correlate of protection. As shown by others (42), the degree of frailty measured by the RFI predicts influenza vaccine effectiveness against hospitalization in older adults. This observation highlights the importance of cell-mediated immune responses when antibodies fail to prevent natural influenza infection and the serious complications of influenza, although it has also been noted that high dose of the same HA/NA only based vaccine can significantly overcome low responsiveness of the vaccine in elderly (43). Further, the direct measure of T cell/cytokine responses to influenza vaccination in the absence of a live influenza virus challenge (*ex vivo*) may not predict protection in older adults. Therefore, frailty may yet influence the outcome of an influenza infection.

Preclinical and clinical biomarkers predictive of vaccine immunogenicity, efficacy, and safety have been extensively studied. The immunoprofiling data in this study showed early plasma cytokine responses induced by the vaccine with a prominent expression of IFN-γ-induced protein precursor 10 (IP-10 or CXCL-10) as early as day 2 after vaccination. High expression of interferon-induced genes, and in particular IP-10, has been shown to be a predictive signature of vaccine efficacy in influenza (44–46), yellow fever (47), and more recently Ebola (48). The data from elderly subjects in this study further supports a pivotal role of IP-10 pathway in anti-viral immune response and chemoattraction of immune cells triggered by vaccination and suggests more investigation of its specific role in vaccine immunogenicity.

To our best knowledge this is the first reported comprehensive study of the immunology of influenza vaccination in the elderly in an Asian cohort. Several limitations of this study have been highlighted including low number of extremely frail elderly, high female to male ratio, year-round circulation of seasonal influenza in Singapore due to its geographical location and climate which might affect pre-existing immunity, and lack of sufficient cell-mediated immune responses by the vaccine. Despite these limitations, in conclusion, the observations from this study support vaccination as a rational strategy for preventing seasonal influenza infection and associated mortality in the elderly.

## Ethics statement

This study was carried out in accordance with the recommendations and approval of Singapore Domain Specific Review Board, National Healthcare Group, Singapore, Ph. +65-64713266 (Approval no. 2012/01214). All subjects gave written informed consent in accordance with the Declaration of Helsinki.

## Author contributions

AL, LV, TN, PT, NBo, and NBu conceived the study. SN and TN recruited volunteers and performed clinical and frailty assessments. PT supervised the Vaxigrip clinical trial. CT, XC, EM, GW, BA, and AL performed Luminex, immunophenotyping, microarray, and serological assays. LV and NBu supervised the antibody titer assays performed at GCI and Sanofi Pasteur. CC analyzed microarray data. SM-S contributed data and analysis on exposure of the cohort to circulating viruses. VN, XC, YL, MP, NBo and AL performed data analysis, wrote and critically reviewed the manuscript and agree to be accountable for its accuracy.

### Conflict of interest statement

CC, NB and LV were employed by Sanofi Pasteur, Marcy-l'Étoile, France, and the author NB was employed by Nestlé Research Cente, Singapore. The remaining authors declare that the research was conducted in the absence of any commercial or financial relationships that could be construed as a potential conflict of interest.
