# Supplementary material for: Influenza Vaccine-Induced Antibody Responses Are Not Impaired by Frailty in the Community-Dwelling Elderly With Natural Influenza Exposure
Source: Front Immunol. 2018 Oct 24;9:2465. doi: 10.3389/fimmu.2018.02465 (PMC6207627; doi:10.3389/fimmu.2018.02465)

Supplementary Material

Influenza Vaccine-Induced Antibody Responses are Not Impaired by Frailty in the Community-Dwelling Elderly with Natural Influenza Exposure

**Vipin Narang, Yanxia Lu, Crystal Tan, Xavier Camous, Ma Shwe Zin Nyunt, Christophe Carre, Esther Mok, Glenn Wong, Sebastian Maurer-Stroh, Brian Abel, Nicolas Burdin, Michael Poidinger, Paul A. Tambyah, Nabil Bosco, Lucian Visan, Tze Pin Ng, Anis Larbi***

***Correspondence:** Anis Larbi: Anis_Larbi@immunol.a-star.edu.sg

Supplementary Figure 1. Deficits included in the computation of Rockwood’s frailty index.

| Sno | Parameter | Scoring |
| --- | --- | --- |
| 1 | Self-rated health | Excellent = 0, Very good = 0.25, Good = 0.5, Fair = 0.75, Poor = 1 |
| 2 | Fall in last one year? | Yes = 1, No = 0 |
| 3 | MOCA score (out of 30) | Score below 26 = 1, Score 26 or above = 0 |
| 4 | Unintended weight loss of 10 pounds (4.5 kg) or more in the past 6 months | Yes = 1, No = 0 |
| 5 | BMI<18.5 or BMI>30 | Yes = 1, No = 0 |
| 6 | Low knee extension (measure of muscle weakness) determined using dominant knee extension in kilograms, taking average over 3 trials | Male BMI < 21.2: Knee extension < 12.0 kg  Male 21.2 ≤ BMI < 23.5: Knee extension < 13.8 kg  Male 23.5 ≤ BMI < 25.9: Knee extension < 15.3 kg  Male BMI ≥ 25.9: Knee extension < 15.7 kg  Female BMI < 21.4: Knee extension < 10 kg  Female 21.4 ≤ BMI < 24.0: Knee extension < 10.3 kg  Female 24.0 ≤ BMI < 26.8: Knee extension < 10 kg  Female BMI ≥ 26.8: Knee extension < 10 kg |
| 7 | Slow walking speed assessed using 6-meter fast gait speed test taking average of 2 measurements | Male height < 1.64m: Slow if time > 6.3 sec  Male height > 1.64m: Slow if time > 5.4 sec  Female height < 1.53m: Slow if time > 6.6 sec  Female height > 1.53m: Slow if time > 5.4 sec |
| 8 | Polypharmacy | Taking ≥ 6 medicines = 1, otherwise = 0 |
| 9 | Bladder (preceding week) | Incontinent = 1, Occasionally = 0.5, Continent = 0 |
| 10 | Mobility (about the house) | Walk with help = 1, Independent = 0 |
| 11 | Using stairs | Dependent = 1, Need help = 0.5, Independent = 0 |
| 12 | Preparing meals | Unable = 1, Able need help = 0.5, Able = 0 |
| 13 | Housework | Unable = 1, Able need help = 0.5, Able = 0 |
| 14 | Doing laundry | Unable = 1, Able need help = 0.5, Able = 0 |
| 15 | Hypertension (HBP) | Yes = 1, No = 0 |
| 16 | Diabetes | Yes = 1, No = 0 |
| 17 | History of stroke | Yes = 1, No = 0 |
| 18 | Heart disease | Yes = 1, No = 0 |
| 19 | History of eye problem | Yes = 1, No = 0 |
| 20 | History of kidney failure | Yes = 1, No = 0 |
| 21 | History of asthma | Yes = 1, No = 0 |
| 22 | History of tuberculosis | Yes = 1, No = 0 |
| 23 | History of arthritis | Yes = 1, No = 0 |
| 24 | History of osteoporosis | Yes = 1, No = 0 |
| 25 | History of hip fracture | Yes = 1, No = 0 |
| 26 | Depression assessed using geriatric depression score (GDS out of 15) | 0 ≤ GDS < 5 = 0; 5 ≤ GDS < 9 = 0.33; 9 ≤ GDS < 12 = 0.67; GDS ≥ 12 = 1 |
| 27 | History of gastrointestinal problem | Yes = 1, No = 0 |
| 28 | History of thyroid problem | Yes = 1, No = 0 |
| 29 | History of cancer | Yes = 1, No = 0 |
| 30 | Other mental disorders | Yes = 1, No = 0 |

Supplementary Figure 2. Antibodies used for immunophenotyping: (a) TruCount immuno-phenotyping of whole blood, (b) immunophenotyping of PBMCs.

| **Antibody** | **Dye** | **Provider** |
| --- | --- | --- |
| CD123 | BV650 | eBioscience |
| CD14 | PerCP | Biolegend |
| CD16 | A700 | Biolegend |
| CD19 | BV786 | BD |
| CD27 | PE | Biolegend |
| CD3 | PE/cy7 | Biolegend |
| CD38 | APC | BD |
| CD4 | BV510 | Biolegend |
| CD45 | PB | Biolegend |
| CD56 | PE/cy5.5 | eBioscience |
| CD62L | APC/cy7 | Biolegend |
| CD66b | FITC | Biolegend |
| CD8 | PE TR | eBioscience |
| HLA-DR | BV605 | Biolegend |

**(a)**

| **Antibody** | **Dye** | **Provider** |
| --- | --- | --- |
| CD4 | PE-Cy5 | Biolegend |
| CD8 | BV711 | BD |
| CD45RO | BUV 395 | BD |
| CD31 | BV605 | Biolegend |
| Vδ1 | FITC | Abcam |
| CD3 | AF700 | BD |
| CD28 | BUV737 | BD |
| CD95 | PE-CF594 | BD |
| Vδ2 | PerCP | Biolegend |
| CTLA4 (CD152) | PE-Cy7 | Biolegend |
| CXCR5 (CD185) | PE | Biolegend |
| CD27 | BV650 | BD |
| PD1 (CD279) | APC-eF780 | eBioscience |
| KLRG1 | APC | BD |
| CCR7 | BV421 | Biolegend |
| CXCR3 (CD183) | BV510 | Biolegend |
| Live/Dead | Blue (DAPI) | Life Technologies |

**(b)**

Supplementary Figure 3. Pre- and post-vaccination HAI titres and seroprotection and seroconversion rates individually across the five components of Fried’s frailty score. P-values of association were computed using Fisher’s exact test for seroprotection/seroconversion rates and using T-test for HAI titres and ratios, and adjusted for multiple testing using Benjamini Hochberg.

| **Strain** | **HAI Responses** | **Weakness (No = 139, Yes = 66)** | | | |
| --- | --- | --- | --- | --- | --- |
|  |  | ***No*** | ***Yes*** | ***p*** | ***p.adj*** |
| A/H1N1 | Pre-vaccination (Day 0) HAI titre, GMT | 14.7 ± 4.5 | 16.6 ± 5.1 | 0.6154 | 1 |
|  | Pre-vaccination Seroprotection rate, no. (%) | 38 (27.3%) | 19 (28.8%) | 0.8682 | 1 |
|  | Post-vaccination (Day 28) HAI titre, GMT | 258.2 ± 4.4 | 260.7 ± 5.4 | 0.9685 | 1 |
|  | Post-vaccination Seroprotection rate, no. (%) | 129 (92.8%) | 62 (93.9%) | 1 | 1 |
|  | Post/Pre-vaccination HAI ratio | 17.6 ± 4.5 | 15.7 ± 6 | 0.6654 | 1 |
|  | Seroconversion rate, no. (%) | 117 (84.2%) | 54 (81.8%) | 0.6906 | 1 |
| A/H3N2 | Pre-vaccination (Day 0) HAI titre, GMT | 56.1 ± 5.8 | 46.3 ± 5.8 | 0.4664 | 1 |
|  | Pre-vaccination Seroprotection rate, no. (%) | 80 (57.6%) | 31 (47%) | 0.1781 | 1 |
|  | Post-vaccination (Day 28) HAI titre, GMT | 1027.8 ± 3.9 | 958.9 ± 4 | 0.7375 | 1 |
|  | Post-vaccination Seroprotection rate, no. (%) | 133 (95.7%) | 66 (100%) | 0.1799 | 1 |
|  | Post/Pre-vaccination HAI ratio | 18.3 ± 5.6 | 20.7 ± 5.8 | 0.6396 | 1 |
|  | Seroconversion rate, no. (%) | 111 (79.9%) | 54 (81.8%) | 0.8511 | 1 |
| B | Pre-vaccination (Day 0) HAI titre, GMT | 65 ± 4 | 71.6 ± 5.9 | 0.6974 | 1 |
|  | Pre-vaccination Seroprotection rate, no. (%) | 96 (69.1%) | 41 (62.1%) | 0.3438 | 1 |
|  | Post-vaccination (Day 28) HAI titre, GMT | 1141.3 ± 3 | 1105 ± 3.2 | 0.8504 | 1 |
|  | Post-vaccination Seroprotection rate, no. (%) | 138 (99.3%) | 66 (100%) | 1 | 1 |
|  | Post/Pre-vaccination HAI ratio | 17.5 ± 4.6 | 15.4 ± 5.4 | 0.5981 | 1 |
|  | Seroconversion rate, no. (%) | 115 (82.7%) | 51 (77.3%) | 0.3487 | 1 |
| **Strain** | **HAI Responses** | **Slowness (No = 124, Yes = 81)** | | | |
|  |  | ***No*** | ***Yes*** | ***p*** | ***p.adj*** |
| A/H1N1 | Pre-vaccination (Day 0) HAI titre, GMT | 15.5 ± 4.7 | 14.9 ± 4.7 | 0.8541 | 1 |
|  | Pre-vaccination Seroprotection rate, no. (%) | 35 (28.2) | 22 (27.2) | 1 | 1 |
|  | Post-vaccination (Day 28) HAI titre, GMT | 264.6 ± 4.3 | 250.7 ± 5.4 | 0.8141 | 1 |
|  | Post-vaccination Seroprotection rate, no. (%) | 116 (93.5) | 75 (92.6) | 0.7844 | 1 |
|  | Post/Pre-vaccination HAI ratio | 17.1 ± 4.6 | 16.8 ± 5.6 | 0.9563 | 1 |
|  | Seroconversion rate, no. (%) | 104 (83.9) | 67 (82.7) | 0.8494 | 1 |
| A/H3N2 | Pre-vaccination (Day 0) HAI titre, GMT | 46.9 ± 5.3 | 63.2 ± 6.6 | 0.2476 | 1 |
|  | Pre-vaccination Seroprotection rate, no. (%) | 66 (53.2) | 45 (55.6) | 0.7756 | 1 |
|  | Post-vaccination (Day 28) HAI titre, GMT | 1076.3 ± 3.6 | 905.1 ± 4.4 | 0.3898 | 1 |
|  | Post-vaccination Seroprotection rate, no. (%) | 122 (98.4) | 77 (95.1) | 0.2157 | 1 |
|  | Post/Pre-vaccination HAI ratio | 22.9 ± 5.6 | 14.3 ± 5.6 | 0.0565 | 1 |
|  | Seroconversion rate, no. (%) | 102 (82.3) | 63 (77.8) | 0.473 | 1 |
| B | Pre-vaccination (Day 0) HAI titre, GMT | 73 ± 4.5 | 59 ± 4.6 | 0.3324 | 1 |
|  | Pre-vaccination Seroprotection rate, no. (%) | 86 (69.4) | 51 (63) | 0.3653 | 1 |
|  | Post-vaccination (Day 28) HAI titre, GMT | 1230.9 ± 3 | 990.2 ± 3.1 | 0.178 | 1 |
|  | Post-vaccination Seroprotection rate, no. (%) | 123 (99.2) | 81 (100) | 1 | 1 |
|  | Post/Pre-vaccination HAI ratio | 16.9 ± 5 | 16.8 ± 4.6 | 0.9785 | 1 |
|  | Seroconversion rate, no. (%) | 101 (81.5) | 65 (80.2) | 0.8569 | 1 |

| **Strain** | **HAI Responses** | **Exhaustion (No = 121, Yes = 84)** | | | |
| --- | --- | --- | --- | --- | --- |
|  |  | ***No*** | ***Yes*** | ***p*** | ***p.adj*** |
| A/H1N1 | Pre-vaccination (Day 0) HAI titre, GMT | 15 ± 4.8 | 15.6 ± 4.6 | 0.8598 | 1 |
|  | Pre-vaccination Seroprotection rate, no. (%) | 34 (28.1%) | 23 (27.4%) | 1 | 1 |
|  | Post-vaccination (Day 28) HAI titre, GMT | 207 ± 4.5 | 357.7 ± 4.8 | 0.0137 | 1 |
|  | Post-vaccination Seroprotection rate, no. (%) | 111 (91.7%) | 80 (95.2%) | 0.4068 | 1 |
|  | Post/Pre-vaccination HAI ratio | 13.8 ± 4.6 | 22.9 ± 5.2 | 0.0275 | 1 |
|  | Seroconversion rate, no. (%) | 99 (81.8%) | 72 (85.7%) | 0.5677 | 1 |
| A/H3N2 | Pre-vaccination (Day 0) HAI titre, GMT | 47.5 ± 5.9 | 61.4 ± 5.7 | 0.3022 | 1 |
|  | Pre-vaccination Seroprotection rate, no. (%) | 61 (50.4%) | 50 (59.5%) | 0.2038 | 1 |
|  | Post-vaccination (Day 28) HAI titre, GMT | 872 ± 4 | 1233.3 ± 3.7 | 0.0705 | 1 |
|  | Post-vaccination Seroprotection rate, no. (%) | 116 (95.9%) | 83 (98.8%) | 0.4041 | 1 |
|  | Post/Pre-vaccination HAI ratio | 18.4 ± 6 | 20.1 ± 5.3 | 0.7133 | 1 |
|  | Seroconversion rate, no. (%) | 95 (78.5%) | 70 (83.3%) | 0.4745 | 1 |
| B | Pre-vaccination (Day 0) HAI titre, GMT | 74.3 ± 4.3 | 58 ± 4.9 | 0.2584 | 1 |
|  | Pre-vaccination Seroprotection rate, no. (%) | 85 (70.2%) | 52 (61.9%) | 0.23 | 1 |
|  | Post-vaccination (Day 28) HAI titre, GMT | 1102.9 ± 3 | 1168.9 ± 3.3 | 0.7199 | 1 |
|  | Post-vaccination Seroprotection rate, no. (%) | 120 (99.2%) | 84 (100%) | 1 | 1 |
|  | Post/Pre-vaccination HAI ratio | 14.9 ± 4.6 | 20.2 ± 5.1 | 0.1775 | 1 |
|  | Seroconversion rate, no. (%) | 98 (81%) | 68 (81%) | 1 | 1 |
| **Strain** | **HAI Responses** | **Low physical activity (No = 137, Yes = 68)** | | | |
|  |  | ***No*** | ***Yes*** | ***p*** | ***p.adj*** |
| A/H1N1 | Pre-vaccination (Day 0) HAI titre, GMT | 17.1 ± 5 | 12.1 ± 4.1 | 0.1199 | 1 |
|  | Pre-vaccination Seroprotection rate, no. (%) | 43 (31.4%) | 14 (20.6%) | 0.1359 | 1 |
|  | Post-vaccination (Day 28) HAI titre, GMT | 261.4 ± 4.8 | 254.4 ± 4.6 | 0.9061 | 1 |
|  | Post-vaccination Seroprotection rate, no. (%) | 126 (92%) | 65 (95.6%) | 0.395 | 1 |
|  | Post/Pre-vaccination HAI ratio | 15.3 ± 5 | 21 ± 4.9 | 0.1832 | 1 |
|  | Seroconversion rate, no. (%) | 111 (81%) | 60 (88.2%) | 0.2338 | 1 |
| A/H3N2 | Pre-vaccination (Day 0) HAI titre, GMT | 52.8 ± 5.8 | 52.7 ± 5.8 | 0.9907 | 1 |
|  | Pre-vaccination Seroprotection rate, no. (%) | 73 (53.3%) | 38 (55.9%) | 0.7672 | 1 |
|  | Post-vaccination (Day 28) HAI titre, GMT | 1004 ± 3.7 | 1007.3 ± 4.4 | 0.9875 | 1 |
|  | Post-vaccination Seroprotection rate, no. (%) | 133 (97.1%) | 66 (97.1%) | 1 | 1 |
|  | Post/Pre-vaccination HAI ratio | 19 ± 6 | 19.1 ± 4.9 | 0.9795 | 1 |
|  | Seroconversion rate, no. (%) | 108 (78.8%) | 57 (83.8%) | 0.4573 | 1 |
| B | Pre-vaccination (Day 0) HAI titre, GMT | 71.8 ± 4.6 | 58.6 ± 4.4 | 0.3655 | 1 |
|  | Pre-vaccination Seroprotection rate, no. (%) | 93 (67.9%) | 44 (64.7%) | 0.7529 | 1 |
|  | Post-vaccination (Day 28) HAI titre, GMT | 1116.6 ± 3.3 | 1156 ± 2.7 | 0.8252 | 1 |
|  | Post-vaccination Seroprotection rate, no. (%) | 136 (99.3%) | 68 (100%) | 1 | 1 |
|  | Post/Pre-vaccination HAI ratio | 15.6 ± 4.9 | 19.7 ± 4.6 | 0.3057 | 1 |
|  | Seroconversion rate, no. (%) | 109 (79.6%) | 57 (83.8%) | 0.5717 | 1 |

| **Strain** | **HAI Responses** | **Weight loss (No = 190, Yes = 15)** | | | |
| --- | --- | --- | --- | --- | --- |
|  |  | ***No*** | ***Yes*** | ***p*** | ***p.adj*** |
| A/H1N1 | Pre-vaccination (Day 0) HAI titre, GMT | 15.2 ± 4.7 | 16.6 ± 4.8 | 0.829 | 1 |
|  | Pre-vaccination Seroprotection rate, no. (%) | 53 (27.9) | 4 (26.7) | 1 | 1 |
|  | Post-vaccination (Day 28) HAI titre, GMT | 254.3 ± 4.8 | 327.5 ± 4.2 | 0.5219 | 1 |
|  | Post-vaccination Seroprotection rate, no. (%) | 176 (92.6) | 15 (100) | 0.6053 | 1 |
|  | Post/Pre-vaccination HAI ratio | 16.8 ± 5 | 19.7 ± 5.2 | 0.7195 | 1 |
|  | Seroconversion rate, no. (%) | 158 (83.2) | 13 (86.7) | 1 | 1 |
| A/H3N2 | Pre-vaccination (Day 0) HAI titre, GMT | 51.6 ± 5.8 | 69.6 ± 5.4 | 0.5178 | 1 |
|  | Pre-vaccination Seroprotection rate, no. (%) | 102 (53.7) | 9 (60) | 0.7895 | 1 |
|  | Post-vaccination (Day 28) HAI titre, GMT | 971.8 ± 4 | 1539.9 ± 2.6 | 0.0995 | 1 |
|  | Post-vaccination Seroprotection rate, no. (%) | 184 (96.8) | 15 (100) | 1 | 1 |
|  | Post/Pre-vaccination HAI ratio | 18.8 ± 5.7 | 22.1 ± 5.8 | 0.7362 | 1 |
|  | Seroconversion rate, no. (%) | 153 (80.5) | 12 (80) | 1 | 1 |
| B | Pre-vaccination (Day 0) HAI titre, GMT | 67.3 ± 4.6 | 65 ± 4.7 | 0.934 | 1 |
|  | Pre-vaccination Seroprotection rate, no. (%) | 127 (66.8) | 10 (66.7) | 1 | 1 |
|  | Post-vaccination (Day 28) HAI titre, GMT | 1116.3 ± 3.1 | 1309.9 ± 2.7 | 0.5634 | 1 |
|  | Post-vaccination Seroprotection rate, no. (%) | 189 (99.5) | 15 (100) | 1 | 1 |
|  | Post/Pre-vaccination HAI ratio | 16.6 ± 4.7 | 20.2 ± 7.2 | 0.7141 | 1 |
|  | Seroconversion rate, no. (%) | 156 (82.1) | 10 (66.7) | 0.169 | 1 |

Supplementary Figure 4. (a) Spearman’s and (b) Pearson’s correlations between frailty measures or their individual components and pre- and post-vaccination HAI titres or their ratios. Correlations with significant unadjusted p-values (p < 0.05) are marked with an asterisk symbol. No p-values were significant after adjustment for multiple testing. HAI titres and ratios were log2 transformed while calculating the correlations.


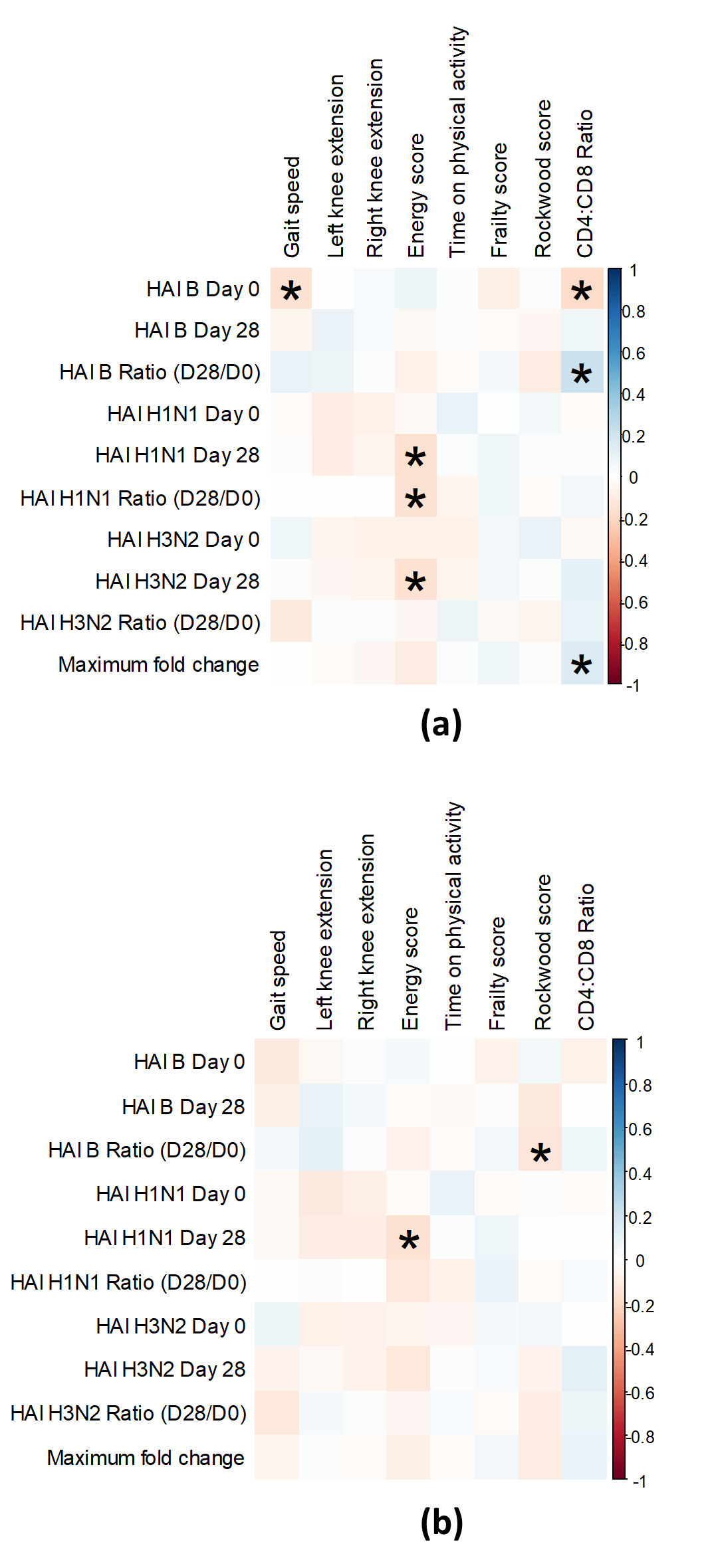


Supplementary Figure 5. Pearson’s correlation (r) and its p-value (p) and linear regression fit (dotted blue line) between Rockwood frailty score and log2 transformed post/pre-vaccination HAI titre ratios for the three vaccinated strains and maximum ratio (maximum fold change) over the three strains. A fold change of four, which marks the threshold for successful seroconversion, is shown by a horizontal dotted red line for comparison with the linear regression fit.


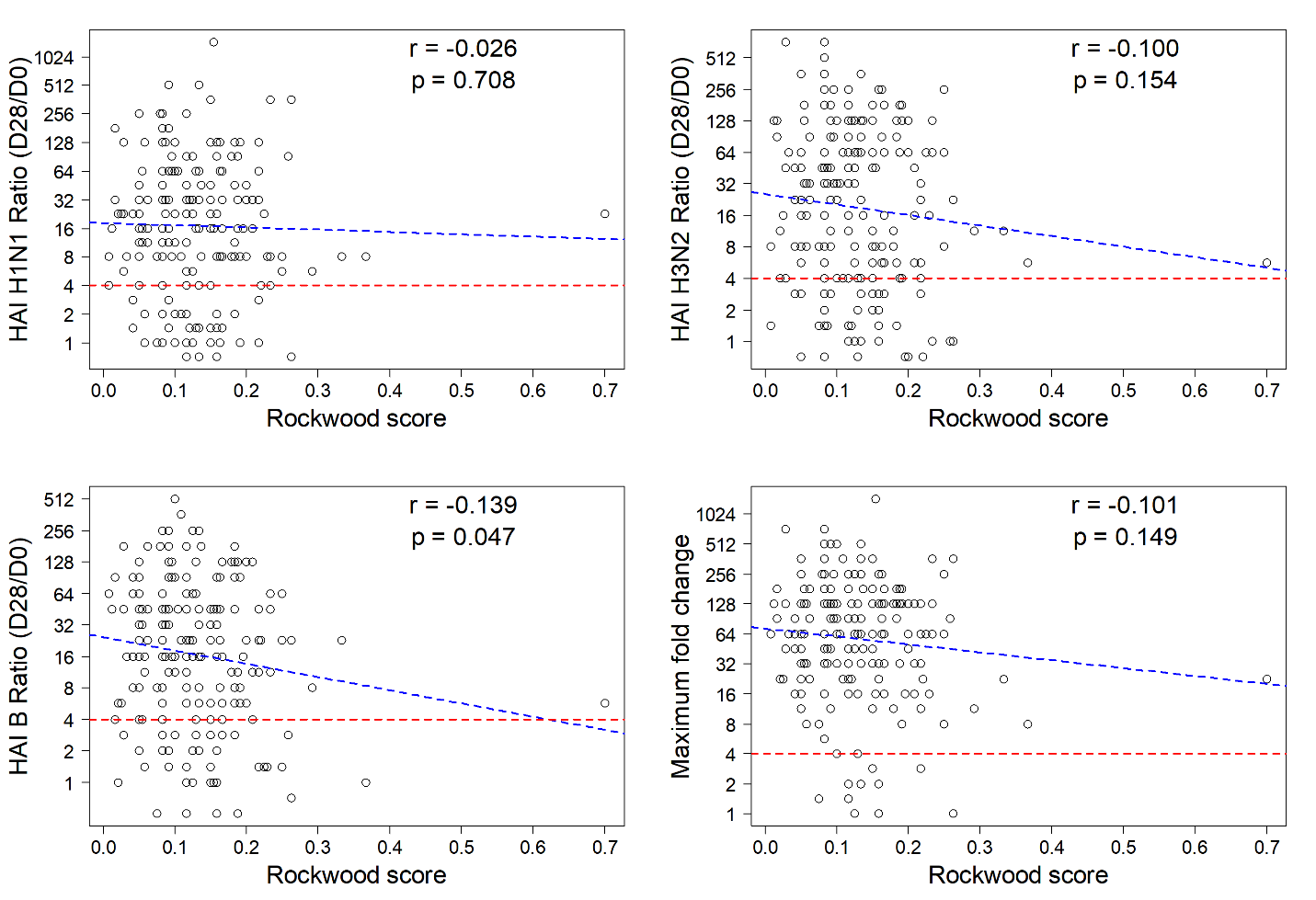


Supplementary Figure 6. Correlation of pre-immunization HAI titre with post-immunization HAI titre and post/pre-immunization titre ratio for each of the three vaccinated strains. Pearson’s correlation, r, its p-value, p, and a linear regression fit (dotted lines) are shown.


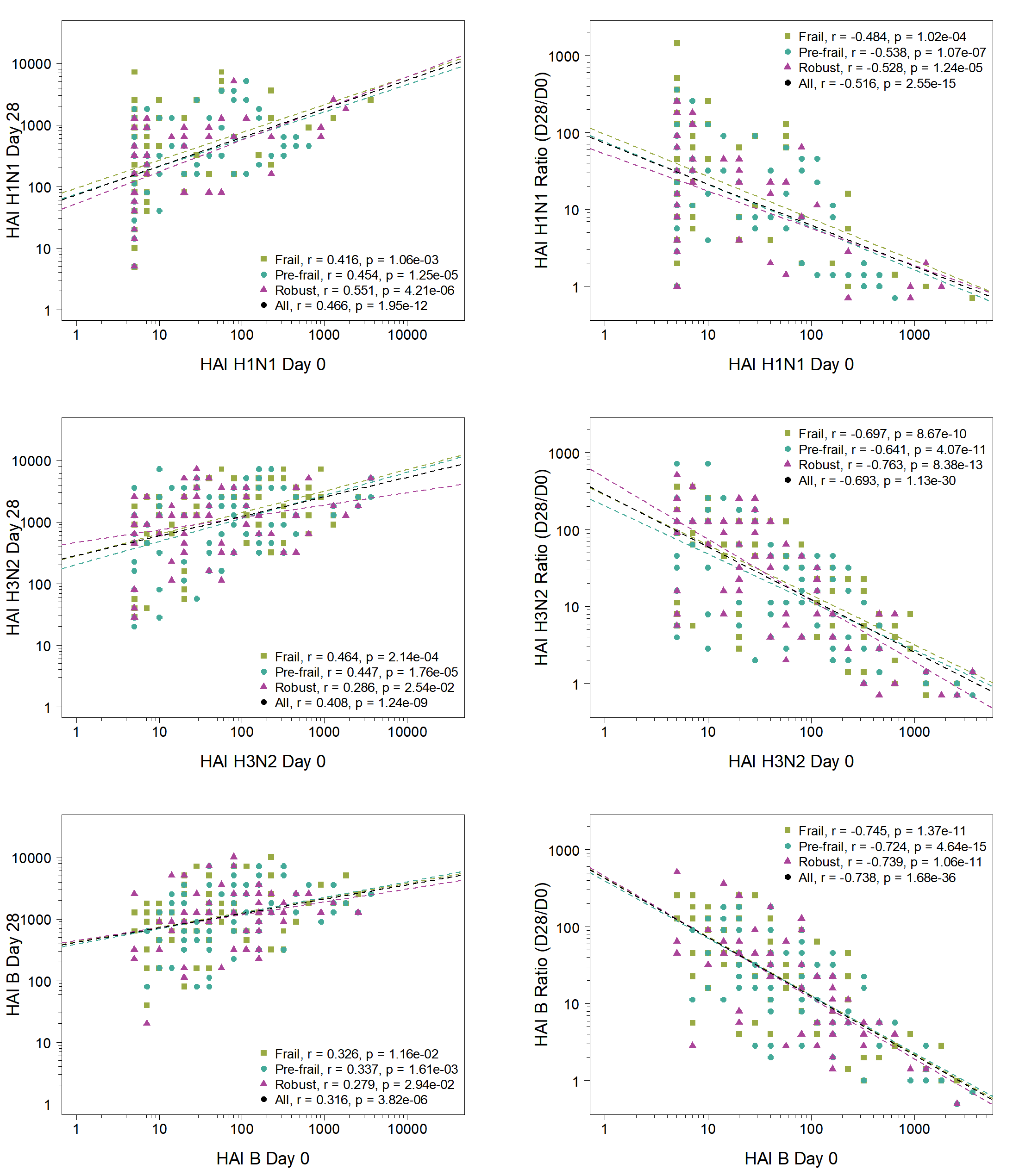


Supplementary Figure 7. Correlation between hemagglutination inhibition (HAI) and microneutralization (MN) titres.


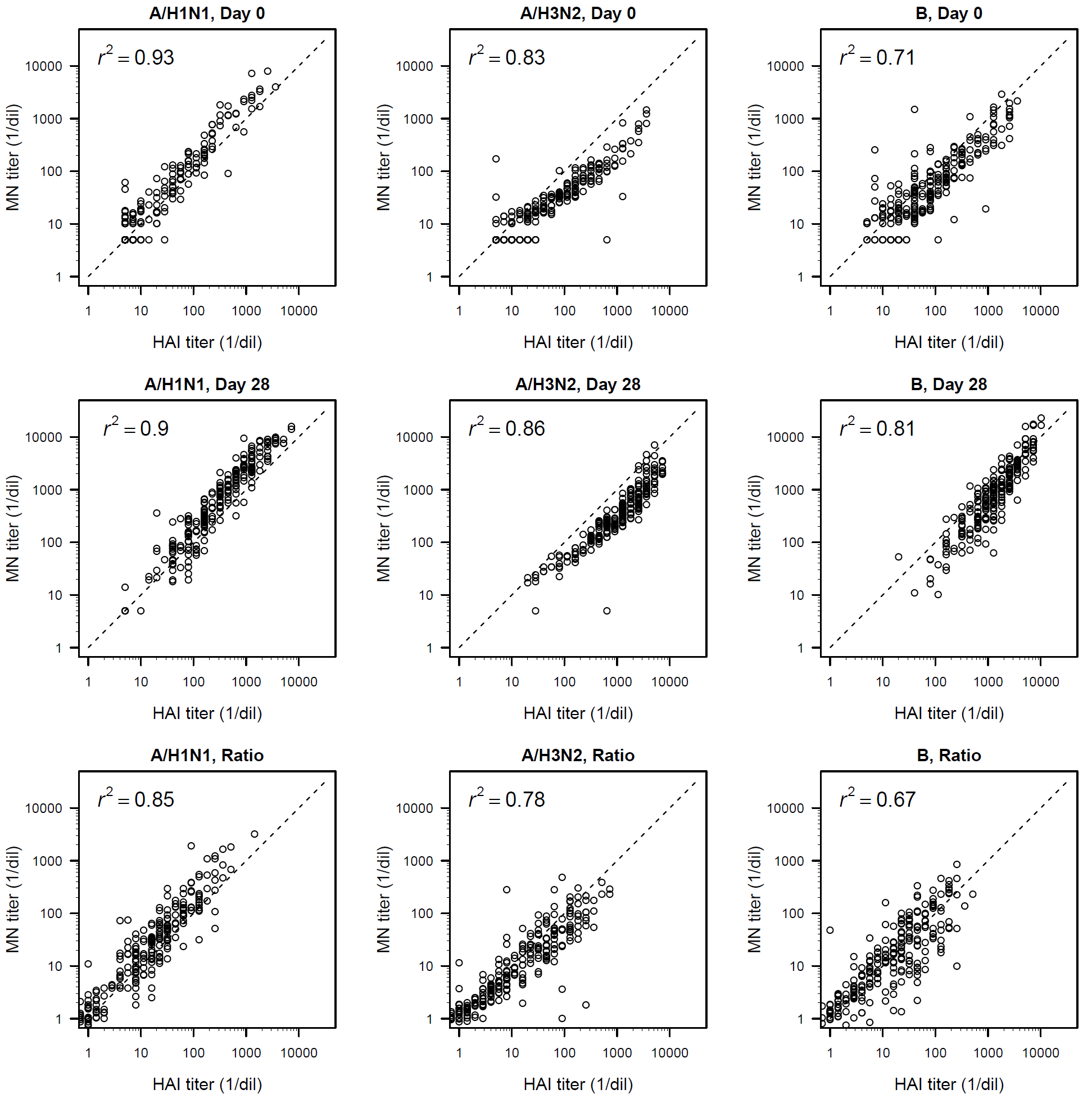


Supplementary Figure 8. Pre-vaccination cellular composition of peripheral blood stratified by frailty. P-values were determined by Mann-Whitney-Wilcoxon U test of medians for unpaired differences.


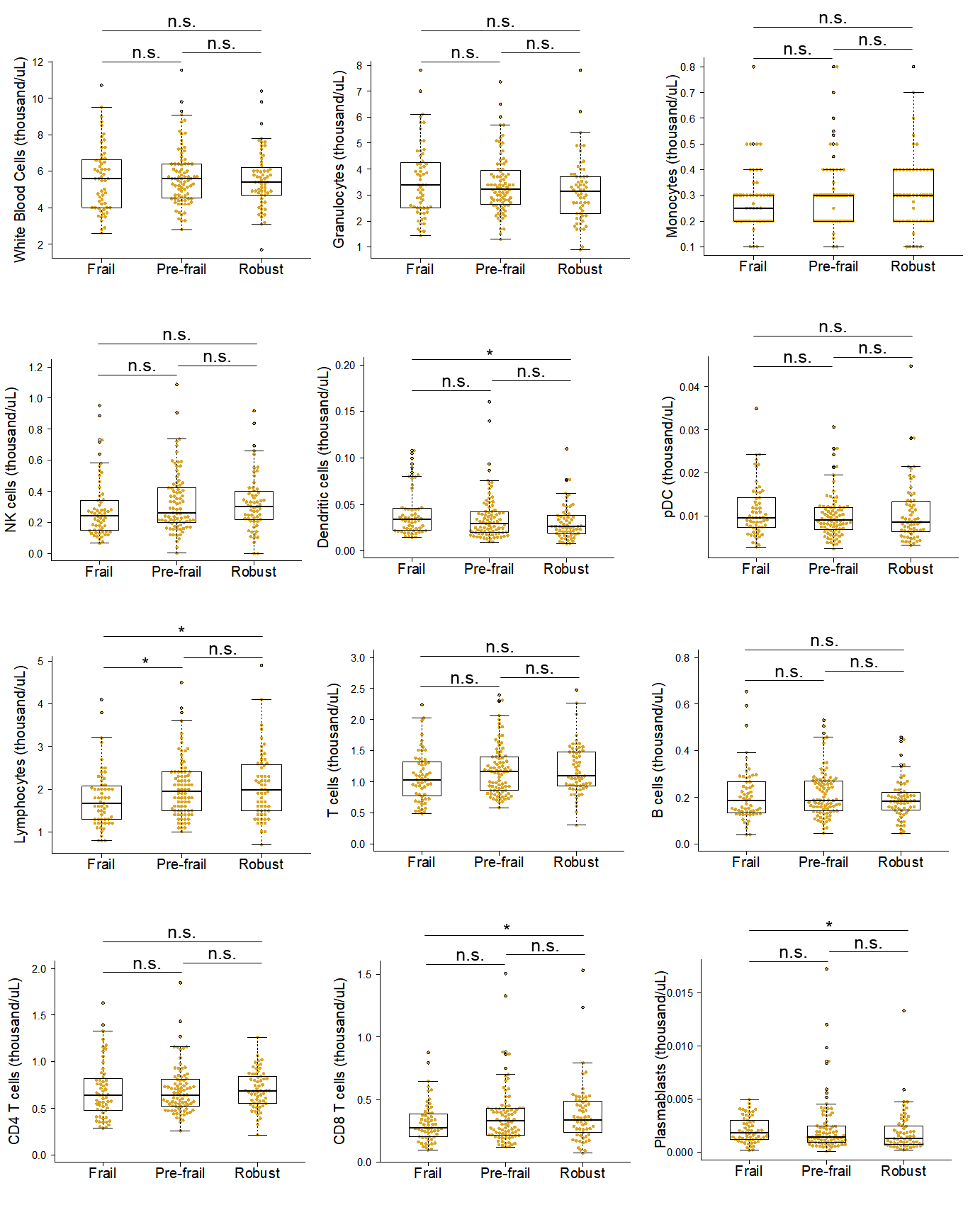


Supplementary Figure 9. Gene ontology analysis of genes that are differently modulated between (a) Pre-frail vs. Frail, and (b) Robust vs. Frail groups comparing Day 28 vs. Day 0 gene expression. Gene ontology analysis was performed at the official website of the gene ontology database (<http://geneontology.org/>) with the choice of GO biological process annotations.


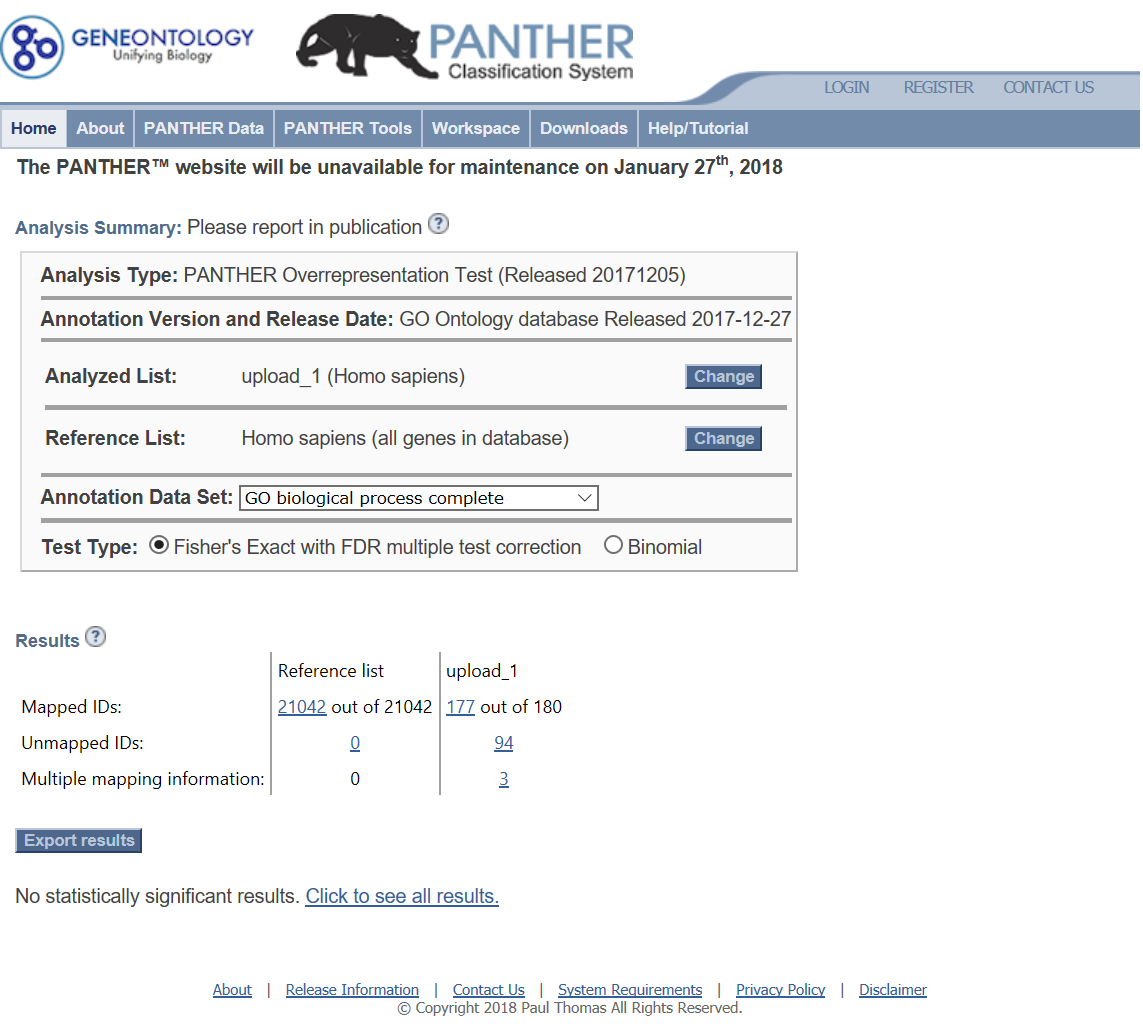


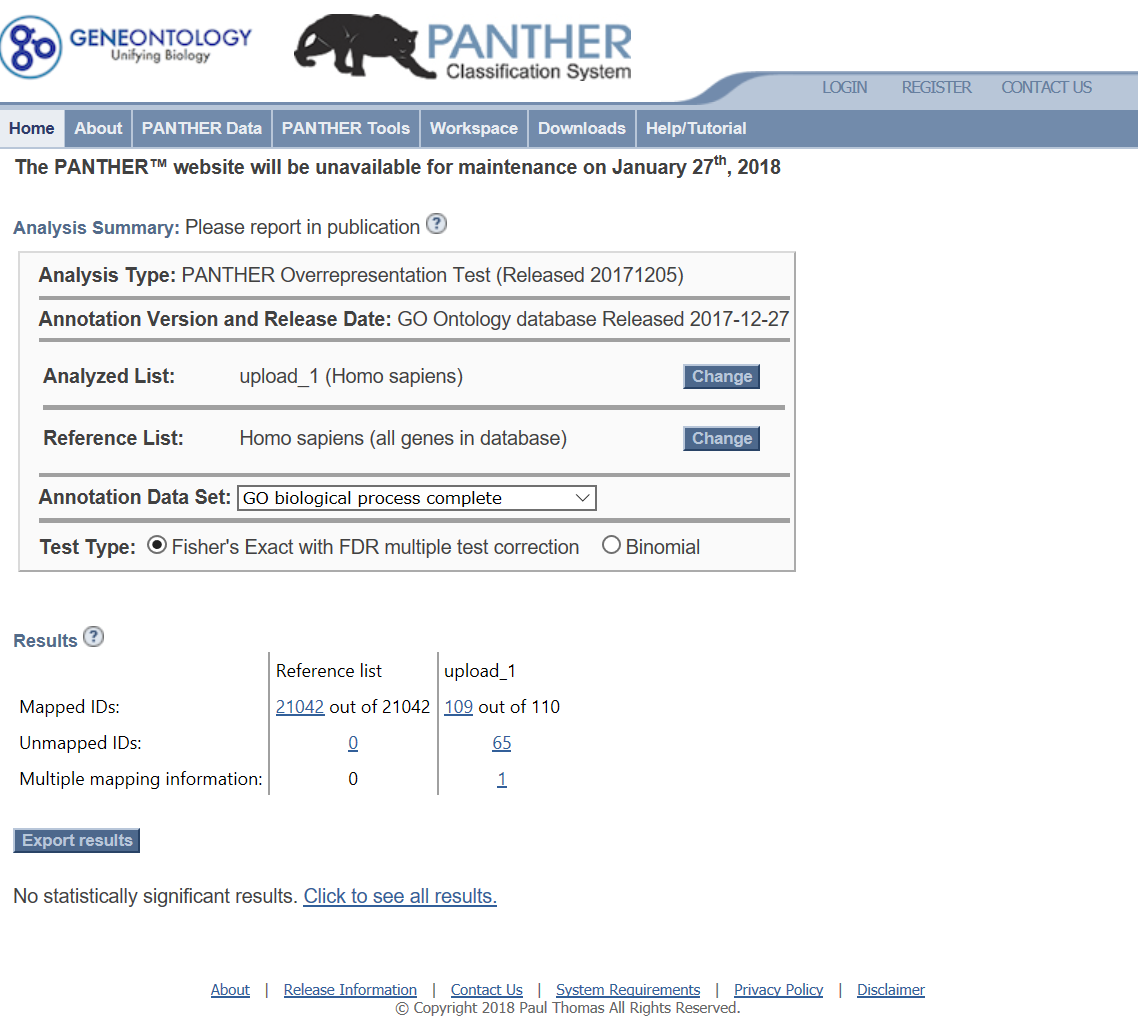


Supplementary Figure 10. Serological status across frailty strata. P-values of association were computed using Fisher’s exact test and adjusted for multiple testing using Benjamini Hochberg.

|  | **All**  **(N = 205)** | **Frail**  **(N = 59)** | **Pre-frail**  **(N = 85)** | **Robust**  **(N = 61)** | ***p*** | ***p.adj*** |
| --- | --- | --- | --- | --- | --- | --- |
| Cytomegalovirus, positive no. (%) | 201 (98) | 58 (98.3) | 82 (96.5) | 61 (100) | 0.3063 | 0.818 |
| Epstein-Barr virus (EA IgG), positive no. (%) | 27 (13.2) | 7 (11.9) | 11 (12.9) | 9 (14.8) | 0.5250 | 0.818 |
| Epstein-Barr virus (EBNA IgG), positive no. (%) | 203 (99) | 58 (98.3) | 85 (100) | 60 (98.4) | 0.5122 | 0.818 |
| H. Pylori, positive no. (%) | 69 (33.7) | 20 (33.9) | 26 (30.6) | 23 (37.7) | 0.8789 | 0.879 |
| Herpes simplex virus Type 1, positive no. (%) | 163 (79.5) | 45 (76.3) | 66 (77.6) | 52 (85.2) | 0.6617 | 0.818 |
| Herpes simplex virus Type 2, positive no. (%) | 39 (19) | 14 (23.7) | 19 (22.4) | 6 (9.8) | 0.1802 | 0.818 |
| Respiratory syncytial virus, positive no. (%) | 11 (5.4) | 5 (8.5) | 6 (7.1) | 0 (0) | 0.1136 | 0.818 |
| Vesicular stomatitis virus, positive no. (%) | 13 (6.3) | 5 (8.5) | 5 (5.9) | 3 (4.9) | 0.5277 | 0.818 |
| Human herpesvirus 6, positive no. (%) | 87 (42.4) | 28 (47.5) | 34 (40) | 25 (41) | 0.6594 | 0.818 |
| Human herpesvirus 8, positive no. (%) | 11 (5.4) | 4 (6.8) | 5 (5.9) | 2 (3.3) | 0.7362 | 0.818 |

**Supplementary Figure 11. (a) Relative proportions of influenza subtypes in Singapore since 2011 as detected by PCR of representative samples as part of national surveillance efforts by the National Public Health Laboratory (NPHL), Singapore, (b) Considering the baseline titre and temporal progression approximated by patient ID number, a comparison between baseline titres and actual subtype prevalence in the respective time period is shown. This suggests that high base (day 0) titres may be mainly due to recent infections by circulating viruses.**


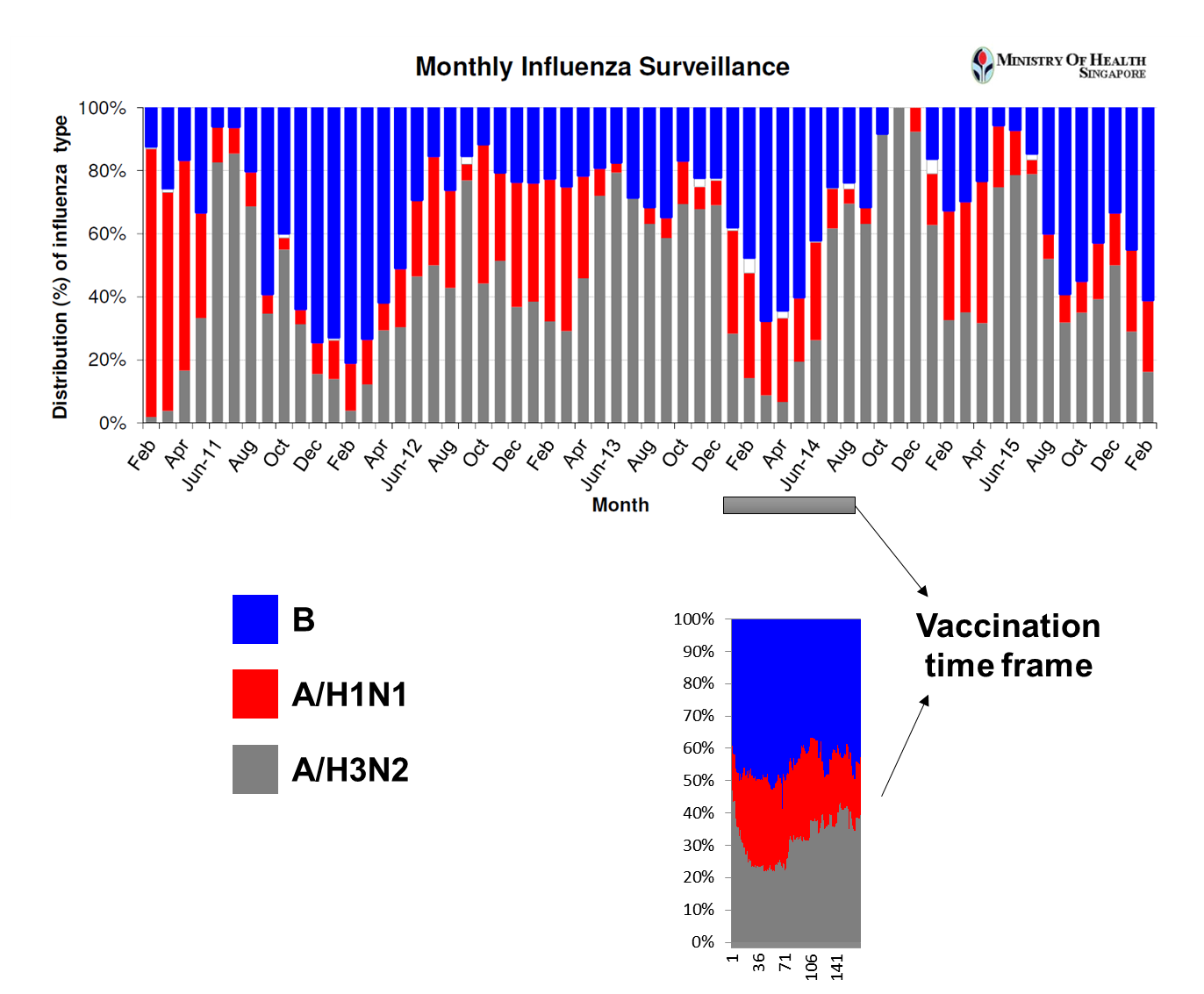


Supplementary Figure 12. Pre- and post-vaccination counts of CD8 T cells in the three frailty groups. P-values were determined by Mann-Whitney-Wilcoxon U test of medians for unpaired differences.


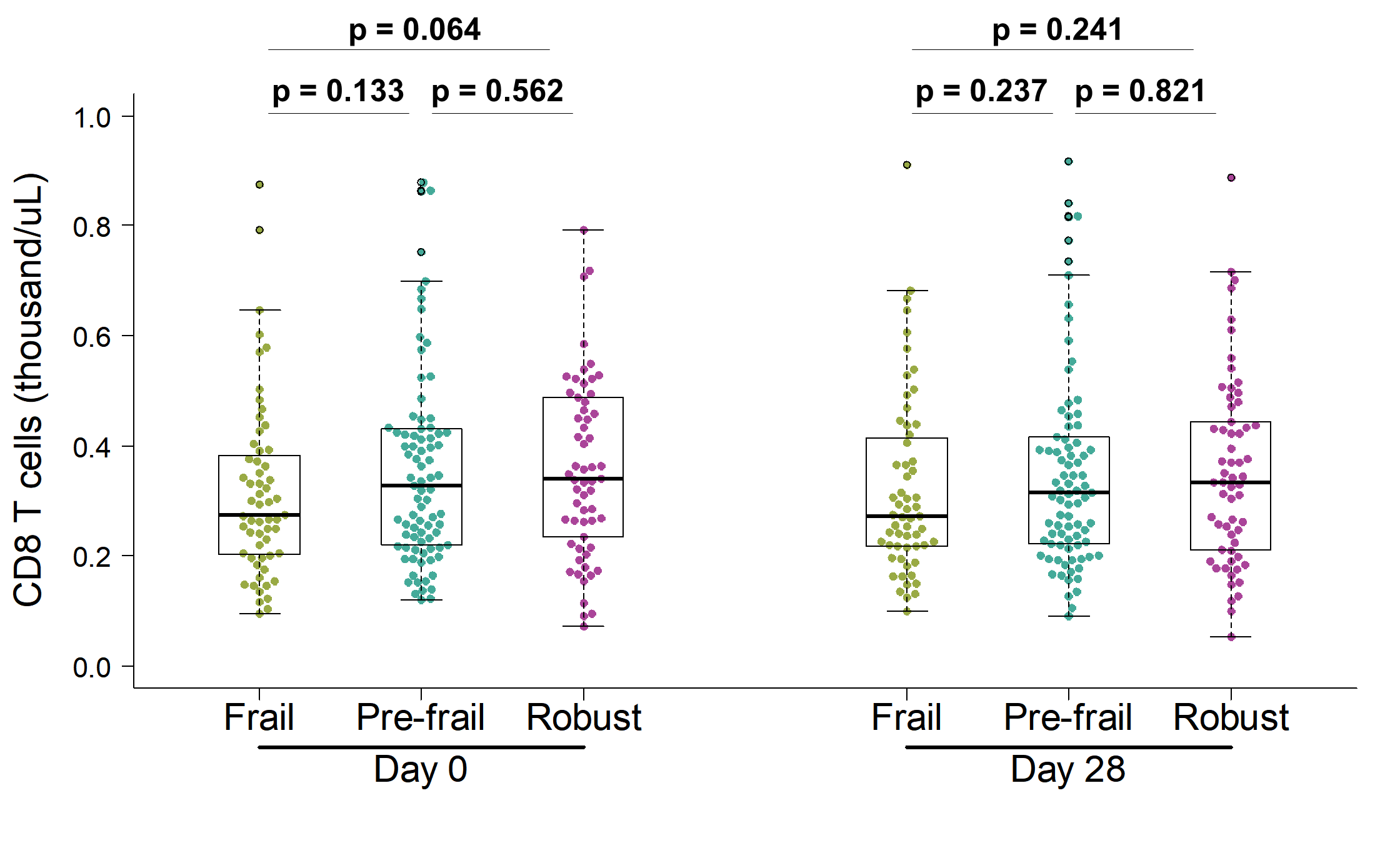

Supplement: Supplementary file 1 [file Data_Sheet_1.docx]
